# Influences of Glyphosate Contaminations and Concentrate Feed on Performance, Blood Parameters, Blood Cell Functionality and DNA Damage Properties in Fattening Bulls

**DOI:** 10.3390/ani13091499

**Published:** 2023-04-28

**Authors:** Ann-Katrin Heymann, Karina Schnabel, Fabian Billenkamp, Susanne Bühler, Jana Frahm, Susanne Kersten, Ulrich Meyer, Dirk von Soosten, Sven Dänicke

**Affiliations:** Institute of Animal Nutrition, Friedrich-Loeffler-Institut (FLI), Federal Research Institute for Animal Health, 38116 Braunschweig, Germany; ann-katrin.heymann@fli.de (A.-K.H.); karinaalster@gmail.com (K.S.); susanne.buehler@fli.de (S.B.); jana.frahm@fli.de (J.F.); susanne.kersten@fli.de (S.K.); ulrich.meyer@fli.de (U.M.); dirk.von_soosten@fli.de (D.v.S.); sven.daenicke@fli.de (S.D.)

**Keywords:** DNA damage, fattening bulls, gene expression, glyphosate residues, hematology, oxidative stress, performance, serum metabolites

## Abstract

**Simple Summary:**

Residues of herbicidal active substances such as glyphosate in ruminant feed lead to oral exposure in animals. Consequently, the possible toxic influences of glyphosate on the health of ruminants are of concern. While some studies have analyzed the effects of glyphosate residues on dairy cows, studies with fattening bulls are scarce. Therefore, the present feeding study on German Holstein bulls was conducted within a real-life in vivo scenario that could occur in Germany before restrictions on glyphosate usage are enacted and might still be realistic in other countries. As well as feeding diets with or without glyphosate residues for several weeks, different concentrate proportions were employed to analyze the putative influences of varying energy and nutrient supplies and consequently different ruminal milieus on potential glyphosate effects. Glyphosate exposure did not adversely affect animals’ performances or other health-related characteristics upon the tested conditions. The observed putative effects of glyphosate on selected blood parameters were rather weak and not consistent. In contrast, concentrate feed and time markedly influenced most of the experimental parameters. In summary, upon the formerly realistic exposure conditions in Germany, all animals remained clinically inconspicuous throughout the trial.

**Abstract:**

Glyphosate (GLY), the active substance in non-selective herbicides, is often found in ruminant feed. The present feeding study aimed to investigate the effects of GLY-contaminated rations and different concentrate feed proportions (CFP) on the health of fattening German Holstein bulls. Bulls were grouped by low (LC) or high (HC) CFP with (GLY_LC_, GLY_HC_) or without GLY-contaminations (CON_LC_, CON_HC_) in their rations. Intakes (dry matter, water) and body weight were documented continuously lasting over an average range from 392.2 ± 60.4 kg to 541.2 ± 67.4 kg (mean ± SD). Blood samples collected at the trial’s beginning, and after 7 and 15 weeks, were analyzed for hematological and clinical-chemical traits, functional properties of leukocytes, redox parameters and DNA damage. The average GLY exposures of 128.6 (GLY_HC_), 213.7 (GLY_LC_), 1.3 (CON_HC_) and 2.0 µg/kg body weight/d (CON_LC_) did not lead to GLY effects for most of the assessed parameters relating to animal health and performance. CFP and time displayed marked influences on most of the experimental parameters such as higher dry matter intake and average daily gain in HC compared with the LC groups. GLY effects were rather weak. However, the observed interactive effects between GLY and CFP and/or time occurring in an inconsistent manner are likely not reproducible. Finally, all animals remained clinically inconspicuous, which brings into question the physiological relevance of putative GLY effects.

## 1. Introduction

Due to its mode of action as a highly effective inhibitor of the enzyme 5-enolpyruvyl-shikimate-3-phosphate synthase, glyphosate (GLY; N-(phosphonomethyl)glycine) is used as an active substance in common non-selective herbicides [1]. In 2017, GLY sales accounted for 33% of the total herbicides sales in Europe, whereas, at a global level, 92% of total herbicide sales were related to GLY [2]. Their intense use in agriculture has led to growing public concerns and controversies in the literature about the possible toxic effects of GLY on human and animal health, as well as the potential environmental impacts [3,4]. GLY residues in livestock feed such as soybean meal can result in up to 0.95 mg GLY/kg dry matter in concentrates and consequently lead to oral exposure in dairy cows [5]. In dairy cows, about 69% of ingested GLY is excreted via feces (61 ± 11%) and urine (8 ± 3%) [5], while GLY concentrations in blood and milk are low [6,7] and often below the limit of detection (LOD) [5,8]. It has been postulated that consumed and non-excreted GLY remainders might be subject to ruminal microbial degradation [5]. Moreover, GLY’s degradation product, aminomethylphosphonic acid (AMPA), was detected in lower levels in the feedstuff, feces and urine of dairy cows, implicating oral exposure with AMPA, the ruminal degradation of GLY to AMPA or a combination of both processes with potential consequences for the health of animals [5,8]. However, GLY residues were reported in the liver of pigs [9], not influencing the absolute and relative liver weights [9], and in the intestine, kidney and muscles of dairy cows [10]. Furthermore, GLY concentrations of 4.62 µg/mL [11] and 2.4 mg/mL [12] were detected in the blood of rats treated with GLY (orally, 400 mg/kg body weight (BW) [11]) or the commercial product Roundup (intraperitoneally, 269.9 mg GLY/kg [12]). GLY might thus affect the hematological and functional properties of blood cells in rather artificially high exposure scenarios. Alterations in hematological parameters were reported for mice treated with 500 mg Roundup Original^®^ (205 mg GLY)/kg BW/d [13] or rats treated with 3.6, 50.4 and 248.4 mg Roundup Original^®^/kg BW for 15 weeks [14], while hematological parameters in dairy cows remained unsuspicious upon oral GLY exposure [7]. Reactive oxygen species (ROS) are primarily produced by neutrophils as potent antimicrobial agents, while additionally functioning as signaling molecules at low levels [15]. However, an excess of ROS can lead to severe DNA damage and subsequent apoptosis [16]. To prevent oxidative stress in cells and neutralize ROS, the antioxidant defense system, including the enzymes superoxide dismutase (SOD) and glutathione peroxidase (GPx), ensures an oxidative balance/normal redox status [16]. Both ROS generation and the antioxidative defense system were reported to be adversely affected by GLY or its formulations. An increased ROS production of human peripheral blood mononuclear cells (PBMC) and erythrocytes was detected after GLY exposure in vitro [17,18,19]. Additionally, the apoptotic potential of GLY was reported [19] and GLY-induced DNA damage in human PBMC were considered as ROS-mediated effects [17].

In contrast to in vitro studies and studies in laboratory animals, the adverse effects of GLY residues were not observed regarding performance [8], ruminal microbiome [20], serum metabolites [6,8], hematology, the functional properties of immune cells, oxidative status and the DNA integrity of blood cells [7] for dairy cows. Dairy cows were exposed to average GLY concentrations of 112.6 and 132.8 µg GLY/kg BW/d in the context of varying concentrate feed proportions (CFP) for 16 weeks. These doses resulted from realistic agricultural conditions following legal restrictions at the time of feedstuff production and feeding trial. However, these data were derived from dairy cows and are accordingly limited to female animals in a state of milk production fed with a corresponding diet. In comparison, data on male cattle, that are often fed fattening diets, are scarce [21]. The present study on German Holstein bulls fed with a fattening diet was conducted similarly to the described studies in dairy cows [6,7,8,20]. It generated comparable data on animal performance, hematological parameters, immune cell subtypes and their functional properties, as well as the oxidative and antioxidative status and DNA integrity of blood. These data were analyzed to test the hypothesis that GLY residues in feedstuffs resulting from permitted herbicide usage in agriculture do not compromise the health of growing Holstein bulls in a scenario reflecting the length of a fattening phase. Like the aforementioned studies on dairy cows, this study represents the real-life GLY exposure conditions before the introduction of stricter restrictions on use in Germany [22]. The diets were designed in the context of different CFPs that meet common agricultural feeding conditions. Based on the resulting differences in fiber, energy and nutrient supply, different ruminal milieus occurred which are of special interest, since in vitro studies suggested the potentially harmful and fiber-content dependent influences of GLY on microbiota in the gastrointestinal tract of dairy cows [23,24]. 

## 2. Materials and Methods

### 2.1. Experimental Design, Feed Production and Sample Collection

The present animal trial was conducted at the experimental station of the Institute of Animal Nutrition, Friedrich-Loeffler-Institut (FLI), in Braunschweig, Germany in accordance with the German Animal Welfare Act and was approved by the Lower Saxony State Office for Consumer Protection and Food Safety (LAVES, 33.19-42502-04-15/1858). A total of 48 German Holstein bulls (BW 392 ± 60.4 kg, age of 332 ± 29 days; mean ± standard deviation) were housed in group boxes (five to six animals per box, two boxes per group) in a thermally non-regulated stable on slatted floor. The animals were weighed immediately after birth and before start of the trial. The animals were blocked by BW and BW gain and randomly assigned to one of four treatment groups. This corresponds to a randomized block design. In a 2 × 2 factorial design, the bulls were fed control rations (CON groups) or GLY-contaminated rations (GLY groups) and additionally, received either low (one kg on fresh matter basis/animal/d, LC groups) or high (HC groups) CFP from an automated feeding system. In HC groups, concentrate amounts were increased from 2.5 kg/animal/d in the first two weeks to 5 kg/animal/d for the following period. To meet the requirements for mineral intake independent of concentrate quantity, individual compositions of concentrates for HC and LC groups were used, respectively. These feeding strategies resulted in four experimental groups (CON_HC_, CON_LC_, GLY_HC_, GLY_LC_) consisting of 12 animals per group. In summary, one group received control rations and high CFP (CON_HC_), one group also received control ration but low CFP (CON_LC_), while the other two groups were fed GLY-contaminated rations and either high CFP (GLY_HC_) or low CFP (GLY_LC_). Until slaughtering, bulls were offered water and roughage for ad libitum consumption while concentrate feed was offered restrictively. Roughage consisting of 79% maize silage and 21% wheat straw based on dry matter was offered in self-feeding stations (Insentec B.V., Marknesse, The Netherlands). Production of feedstuff was conducted on the acreage of the experimental station of the FLI Braunschweig as described previously [8]. Briefly, maize was grown without GLY application, while a part of wheat and peas was treated with Roundup Rekord^®^ (007525–60/MOT, Monsanto, Agrar Deutschland GmbH, Düsseldorf, Germany) as water-soluble granulate with GLY as an active ingredient (720 g GLY/kg GLY solution). Plants were treated pre-harvest with 2.5 L/ha (wheat) and 2 L/ha (peas) according to legislation applied at this time. The GLY-treated wheat and peas were fed as parts of roughage (wheat straw) and concentrates (wheat kernels and peas) to GLY groups, while CON groups received non-contaminated wheat and peas, respectively. 

Feed samples were collected once (straw), twice (concentrates) or five times (maize silage) during the 15 weeks of the trial. Animals were weighed weekly with a continuous scale, while daily water and feed intake, for both concentrates and roughage, of each bull were recorded automatically. After two weeks, one bull (GLY_HC_) was excluded from the trial due to reasons not related to this experiment, while 47 bulls completed the entire trial and were slaughtered within two periods of two weeks after 15 weeks and after 17 weeks evenly distributed over the experimental groups. After slaughtering, organ weights were recorded. Additionally, urine of each bull was sampled and stored at −20 °C for further analyses.

For calculations of individual intakes (dry matter, energy, water, GLY, AMPA) and related parameters, such as average daily gain, only data collected upon slaughtering of the first bulls (week 15) were used to maintain the balanced distribution within the groups. Additionally, data were merged into two periods. Average daily gain during 15 weeks was calculated by dividing differences in BW by time [25]. Data of dry matter intake (DMI) and average daily gain were used for calculations of feed conversion and energy utilization representing MJ metabolizable energy per kg growth through dividing DMI or respective energy intake by average daily gain [25].

Blood samples of all animals were collected before feeding the experimental rations (week 0), at week 7 and at week 15 from a vena jugularis externa using serum tubes and tubes containing sodium-heparin or ethylenediaminetetraacetic (EDTA). 

### 2.2. Feed Samples and Glyphosate Analyses 

Roughage components and concentrates were analyzed for dry matter, crude ash, crude protein, ether extract, crude fiber, neutral detergent fiber, acid detergent fiber, starch and sugar according to the standard methods of Verband Deutscher Landwirtschaftlicher Untersuchungs- und Forschungsanstalten (VDLUFA) [26]. Feed samples were analyzed for GLY and AMPA concentrations by an accredited laboratory (Wessling GmbH, Altenberge, Germany) using liquid chromatography–tandem mass spectrometry (LC–MS/MS) as described in [8]. The limits of quantification (LOQ) for GLY and AMPA were 0.02 and 0.007 mg/kg feed [8]. The components and the chemical composition of roughage and concentrates are shown Table 1. The roughage part of the ration consisted of 79% maize silage and 21% straw on dry matter basis. GLY and AMPA concentrations in feed samples were calculated using concentrations in straw, maize silage and peas in accordance with [8]. Data of fresh matter intake and chemical analyses were combined to calculate individual intakes and consequently GLY exposures.

Additionally, GLY concentrations in urine samples of all animals were measured by an accredited laboratory (Medizinisches Labor Bremen, Bremen, Germany) using gas chromatography–mass spectrometry (**GC–MS**). LOQ for GLY and AMPA were 0.1 µg/L urine.

### 2.3. Analysis of Serum Metabolites

Serum tubes were centrifuged (Heraeus Varifuge 3.0R Heraeus, Osterode, Germany; 2123× *g*, 15 °C, 15 min) and aliquots of serum samples were stored at −80 °C until analysis. Serum concentrations of alkaline phosphatase, aspartate aminotransferase (**AST**), γ-glutamyltransferase (**GGT**), glutamate dehydrogenase (**GLDH**), total protein, total bilirubin, albumin, cholesterol, non-esterified fatty acids (**NEFA**), triglycerides, glucose, β-hydroxybutyrate (**BHB**), urea and uric acid were measured using an automated analyzer (Eurolyser^®^, Type VET CCA, Eurolyser Diagnostica GmbH, Salzburg, Austria).

### 2.4. Hematological Evaluation

Total and differential white and red blood cell counts were measured in EDTA-blood samples using an automatic analyzer (Celltac-α, MEK-6450, Nihon Kohden, Qinlab Diagnostik, Weichs, Germany). Red blood profile included red blood cell count (erythrocytes), hemoglobin concentration, hematocrit, mean corpuscular volume, mean corpuscular hemoglobin, mean corpuscular hemoglobin concentration, red cell distribution width as well as the platelet related parameters mean platelet volume and platelet distribution width. White blood cells (leukocyte), lymphocyte, granulocyte (including basophils and neutrophils), eosinophils and monocyte counts were considered for white blood profile. Results were expressed as total number or as proportion of respective cell type. 

### 2.5. Antioxidant Enzyme Activities and Ferric Reducing Ability of Plasma

For analyzing the ferric reducing ability of plasma (**FRAP**), EDTA-blood was centrifuged (Heraeus Varifuge 3.0R Heraeus, Osterode, Germany; 2123× *g*, 15 °C, 15 min) and plasma was stored at −80 °C until analysis. The FRAP-assay was used to measure the anti-oxidative capacity of plasma according to Benzie and Strain [27]. Antioxidative reactions were photometrically measured for 15 min at 593 nm (Tecan infinite M 200, Grödig, Austria). For determining the enzyme activities, erythrocyte lysate was prepared from 2 mL EDTA-blood mixed with 10 mL cold distilled water. After 10 min centrifugation (10,000× *g*, 4 °C), the supernatant was stored at −80 °C until analysis. Activities of SOD and GPx were measured using Ransod superoxide dismutase- and Ransel glutathione peroxidase assay (Randox Laboratories, Crumlin, UK) in duplicates according to the manufacturer’s protocol and adjusted as described [28]. Hemoglobin concentration of erythrocyte lysate was measured by automatic analyzer (Celltac-α, MEK-6450, Nihon Kohden, Qinlab Diagnostik, Weichs, Germany) and was used for normalization of the enzyme data.

### 2.6. DNA Damage 

After 15 weeks of GLY exposure, DNA damage was detected in individual blood leukocytes by the gel electrophoresis based comet-assay as described in [29] with modifications [7]. In short, EDTA whole blood mixed with 0.5% low melting point agarose (NuSieve Ag, Lonza, Basel, Switzerland) was layered on glass slides on top of a 1.5% medium electroendoosmosis (MEEO) agarose base layer (Karl Roth, Karlsruhe, Germany). After electrophoresis, neutralization and drying of the slides, DNA was stained with DAPI solution (Roti-Mount FluorCare, Karl Roth, Karlsruhe, Germany). Documentation of 100 nuclei per slide (in triplicate) was conducted using fluorescence microscopy (Leica DMI 6000b, Leica Microsystems CMS GmbH, Wetzlar, Germany). DNA damage was evaluated using CASPlab [30]. The percentage of DNA in the tail in relation to total DNA content of the cell was used as indicator for damaged DNA [%] [31]. Additionally, the Olive tail moment, which is the product of tail DNA [%] and the differences between head and tail means were calculated [31].

### 2.7. Flow Cytometric Analysis

Functional parameters for polymorphonuclear leukocytes (PMN) and PBMC were analyzed in whole blood samples using flow cytometry (FACS Canto II, BD Biosciences, San Jose, CA, USA). Respective cells were defined and gated according to their granularity and size based on forward and side scatter measurements. FACS Diva software 6.1.3. (BD Biosciences, San Jose, CA, USA) was used for evaluation of at least 10,000 cells in a standardized manner. Unless otherwise stated, results were expressed as proportions or mean fluorescence intensities (MFI).

### 2.8. T-Cell Phenotyping

Phenotyping of lymphocytes was conducted by double-staining EDTA whole blood samples with monoclonal antibodies for CD4+ (mouse anti-bovine CD4:FITC (fluorescein isothiocyanate)) and CD8+ (mouse anti-bovine CD8:RPE (Phytoerythrin)) or their related isotype controls (mouse IgG2a negative control RPE and mouse IgG2b negative control FITC). All antibodies used were purchased from Bio-Rad, Hercules, CA, USA. Following 30 min of incubation in the dark, lysis of erythrocytes was conducted with lysis buffer (BD Biosciences, San Jose, CA, USA) for 10 min at room temperature. After centrifugation (200× *g*, 5 min, 4 °C) and resuspension in HEPES (4-(2-hydroxyethyl)-1-piperazineethanesulfonic acid)-buffered saline (HBS), samples were measured using flow cytometry as described above. To correct the spillover of fluorochrome emissions, a compensation was performed using BD FACS Diva^TM^ software, version 9.0.1 (BD Biosciences, San Jose, CA, USA).

### 2.9. Intracellular Production of Reactive Oxygen Species

The method for analyzing the ability of blood PMN to produce intracellular ROS was based on oxidation of the non-fluorescent dihydrorhodamine 123 (**DHR**) to the fluorescent metabolite rhodamine 123 (R123+) by free radicals. Whole blood samples were incubated (15 min at 37 °C) with 40 µM DHR solution (Molecular Probes, Eugene, OR, USA) alone to assess the basal (unstimulated) ROS level or with 30 µM TPA (12-O-tetradecanoylphorbol-13-acetate; Sigma-Aldrich, Taufkirchen, Germany) to induce an oxidative burst (stimulated). After an incubation in lysis buffer (BD Pharm Lyse^TM^; BD Biosciences, San Diego, CA, USA) for 10 min in the dark, resulting in lysis of erythrocytes and cell fixation, samples were centrifuged (250× *g*, 5 min, 4 °C) and washed with HBS before flow cytometric measurement in duplicates. Results were expressed as the percentage of R123+ PMN and their MFI, representing the conversion of DHR per cell.

### 2.10. Phagocytosis

Phagocytic activity of PMN was determined using the Phagotest^TM^ kit (Glycotope Biotechnology GmbH, Heidelberg, Germany) according to manufacturer’s protocol and as previously described [7,32]. In short, heparinized blood samples were incubated with FITC-labelled *E. coli* (10 min, 37 °C), quenched, washed and stained with propidium iodide. Samples were measured regarding the proportions of cells ingesting bacteria and the individual phagocytic capacity in duplicates within 60 min after staining using flow cytometry (FACS Canto II, BD Biosciences, San Jose, CA, USA).

### 2.11. Apoptosis

After 15 weeks of GLY exposure, apoptotic PBMC and PMN were determined in whole blood samples using the FITC Annexin V-Apoptosis Detection Kit II (BD Pharmingen^TM^, BD Biosciences, San Diego, CA, USA) according to manufacturer´s protocol and as described in [7]. In brief, samples were incubated with propidium iodide and FITC-coupled annexin allowing the differentiation between early and late apoptotic stages of cells. Including a non-stained control, samples were analyzed in duplicates by flow cytometry (FACS Canto II, BD Biosciences, San Jose, CA, USA). Results were expressed as percentages of doubled-stained cells (FITC Annexin V positive, propidium iodide positive) characterizing late apoptotic cells and single-stained cells representing early apoptotic cells (FITC Annexin V positive, propidium iodide negative). Additionally, the MFI of stained cells was recorded for relative quantification of the annexin binding.

### 2.12. Ex Vivo Proliferation Assay

For the isolation of PBMC, heparinized blood samples were diluted 1:1 with phosphate-buffered saline, and density gradient centrifugation using Biocoll separation solution (Biochrome GmbH, Berlin, Germany) was conducted as described in [33]. Viability and Concanavalin A-stimulated cell proliferation of isolated PBMC was evaluated using Alamar Blue assay as described in [34]. In brief, 1 × 10^5^ cells/200 µL RPMI medium were seeded per well in 96-well plates with or without 2.5 µg/mL Concanavalin A (Sigma Aldrich, Steinheim, Germany). After incubation of plates for 69 h at 37 °C and 5% CO_2_, alamar blue (Bio-Rad Laboratories GmbH, Feldkirchen, Germany) was added in a ratio of 1:10 to each well. After further incubation of 2.5 h at 37 °C, fluorescence of resorufin, resulting from the reduction of the non-fluorescent resazurin by metabolic active cells, was measured at 540 nm (excitation) and 590 nm (emission, Tecan infinite M 200, Grödig, Austria). The stimulation index expressing the in vitro proliferation of PBMC was calculated by dividing the fluorescence of Concanavalin A-stimulated PBMC and non-stimulated PBMC.

### 2.13. Gene Expression Analysis 

Leukocytes’ gene expression was analyzed in order to further investigate observed effects in the comet assay. For this reason, genes involved in DNA repair mechanisms like non-homologous end joining, homologous recombination and base excision repair, as well as ROS and apoptosis-related genes, were chosen (Table 2). For lysis of erythrocytes, 2 mL EDTA blood was subjected to a water lysis procedure using water, 8.8% NaCl, an incubation and three centrifugation steps, as described in [35]. RNA of the final pellet was isolated using the NucleoSpin^®^ RNA extraction kit (Macherey-Nagel GmbH & Co. KG, Düren, Germany) according to the manufacturer’s protocol and to [35]. Afterwards, isolated RNA was frozen in liquid nitrogen and stored at −80 °C until cDNA was synthesized using 1000 ng RNA and the qScript™ cDNA Synthesis Kit (Quanta Biosciences™, Inc., Gaithersburg, MD, USA) according to the manufacturer’s protocol. Before quantitative real time polymerase chain reaction (qRT-PCR), cDNA was diluted 1:10 and stored at −20 °C, and 4.5 µL (representing 22.5 ng of the respective RNA) were used in qRT-PCR. Primer-Blast [36], Beacon Designer (Free Edition Premier biosoft) and Primer3 version 4.0.0 [37,38] were applied for design of gene-specific primer pairs. Primer selection and qRT-PCR conditions are described in detail in [35]. Information about applied primers is shown in Table 2. PCR products were sequenced for quality control. Normalized gene expression data were obtained by CFX Maestro™ 1.1 (Bio-Rad Laboratories, Inc., Hercules, CA, USA) using three reference genes (*UXT*, *UCHL5* and *RPS9*) showing a mean stability M value of 0.233. Additionally, primer specific efficiencies were taken into consideration. While statistical analysis was performed on log transformed gene expression data, charts contain back transformed data for better interpretation.

### 2.14. Statistical Analyses

For statistical analyses, unless stated otherwise, measured parameters were analyzed using the MIXED procedure in SAS version 9.4 (SAS Institute Inc., Cary, NC, USA) with the restricted maximum likelihood method (reml). Treatment (GLY; GLY or CON diet), CFP (HC or LC diet) and time (t; experimental weeks), as well as their interactions (CFP×*t*, GLY×CFP, GLY×*t*, GLY×CFP×*t*) were used as fixed factors and the individual animal was included as random effect. Time was treated as a repeated measurement. For blood sampling data, values at the beginning of the trial (week 0) were used as covariate to account for possible initial differences, if the first sampling point had a significant influence. For each parameter, covariance structure (variance components (vc), compound symmetry (cs), autoregressive (AR(1)) or unstructured (un) covariance structure) was chosen based on the smallest Akaike information criterion [39]. For data cumulated to or measured at only one time point (average daily gain, feed conversion, energy intake and utilization, slaughtering data, data of apoptosis, comet assay and gene expression) the model was reduced to GLY, CFP and GLY×CFP as fixed factors. Unless otherwise stated, values are shown as Least square (LS) means. For calculating Spearman’s rank correlation between parameters, R Studio version 1.2.5042 [40] in R 4.0.3 [41] was used. Results were declared as significant when *p* < 0.05. All figures were created by using R packages ggplot2 [42], ggpubr [43], gridExtra [44], gtable [45], Corrplot [46] with RStudio version 1.2.5042 [40], R 4.0.3 [41].

### 2.15. Data Availability

A detailed overview of numbers of animals used for statistical analyses of parameters, when differing from full data set, is provided in Appendix A, Table A1.

## 3. Results

### 3.1. Glyphosate Exposure, Fattening and Slaughter Performance

In feed, concentrations of GLY in concentrates of GLY_HC_ were higher (0.41 mg/kg dry matter) than in concentrates of GLY_LC_ (0.31 mg/kg dry matter), while GLY concentrations were 0.02 and 0.01 mg/kg dry matter in the respective CON groups. However, used feedstuffs originated from the same batch as fed in [8]. The straw included showed 61.81 mg GLY and 0.59 mg AMPA residues/kg dry matter and displayed the highest residue concentrations of all the feed components. With regard to exposure levels, GLY exposure varied between 47.4 and 70.9 mg/d for group GLY_HC_ and between 75.3 and 109.4 mg/d for group GLY_LC_, corresponding to the average daily GLY exposures of 59.8 mg/d (128.6 µg/kg BW/d) and 92.4 mg/d (213.7 µg/kg BW/d), respectively. GLY exposure for the CON groups was below 1 mg/d (CON_HC_: 0.6 mg/d, 1.3 µg/kg BW/d; CON_LC_: 0.9 mg/d, 2.0 µg/kg BW/d, p_GLY×CFP_ < 0.001, Table 3). The respective average AMPA-exposures in the experimental groups amounted to 0.6 mg/d (GLY_HC_, 1.2 µg/kg BW/d), 0.8 mg/d (GLY_LC_, 1.9 µg/kg BW/d) and 0 mg/d (CON groups, p_GLY×CFP_ < 0.001, Table 3). DMI, DMI-associated energy intake and average daily gain were significantly increased in HC groups compared with the LC groups irrespective of GLY (p_CFP_ < 0.001), while water intake was not affected by any treatment. The opposite pattern was observed for feed conversion (p_CFP_ < 0.001) and energy utilization (p_CFP_ = 0.001) with elevated levels in the LC groups in comparison with the HC groups, irrespective of GLY (Table 3).

Analyses of GLY residues in the urine of the animals before slaughtering varied between 53.2 and 343.5 µg/L for group GLY_HC_ and between 22.5 and 292.5 µg/L for group GLY_LC_ resulting in average concentrations of 148.1 µg/L (GLY_HC_) and 175.1 µg/L (GLY_LC_), while the average GLY concentration in urine of the CON groups were 1.8 µg/L in CON_HC_ and 2.3 µg/L CON_LC_ (p_GLY_ < 0.001, Table 3). The average AMPA concentrations in urine were 10.0 (GLY_HC_), 13.2 (GLY_LC_), 0.3 (CON_HC_) and 0.6 (CON_LC_) µg/L (p_GLY_ < 0.001, Table 3). 

After adjusting to 100 kg BW, the weights of the heart (p_CFP_ = 0.014), liver (p_CFP_ < 0.001), lung (p_CFP_ = 0.029), spleen (p_CFP_ = 0.002), pancreas (p_CFP_ = 0.027), thymus (p_CFP_ = 0.002), thyroid glands (p_CFP_ = 0.009) and prostate gland (p_CFP_ = 0.027) were significantly higher in the HC groups, whereas the weight of the gall bladder was significantly higher in the GLY groups (p_GLY_ = 0.046, Table 3). The weights of the spinal cord, kidneys, tongue, testicles and urinary bladder remained unaffected by the experimental treatments (Table 3). The BW of bulls before slaughtering varied between a minimum individual weight of 385.5 kg (CON_LC_) and a maximum individual weight of 674 kg (CON_HC_) and was significantly higher in the HC groups than in the LC groups irrespective of GLY (p_CFP_ < 0.001, Table 3).

### 3.2. Blood Parameters

#### 3.2.1. Serum Metabolites 

**Figure 1:**
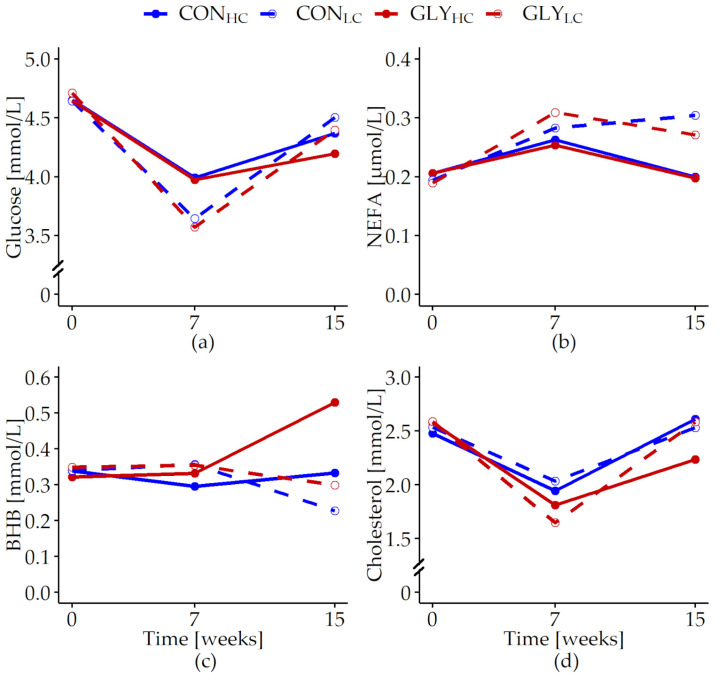
Influences of glyphosate residues and different concentrate feed proportions on serum metabolites. Serum glucose (**a**), NEFA (**b**), BHB (**c**) and cholesterol (**d**) of bulls fed with glyphosate-contaminated (GLY groups, red lines) or control (CON groups, blue lines) rations and with low (LC, 1kg/animal/d, dashed lines) or high (HC, 2.5–5 kg/animal/d, solid lines) concentrate feed proportions (CFP) for 15 weeks (CON_HC_, *n* = 12; CON_LC_, *n* = 12; GLY_HC_, *n* = 11; GLY_LC_, *n* = 12). Values are represented as LS means. Parameters were analyzed with values of week 0 as covariate. Significant *p*-values for glucose (p_CFP×*t*_ < 0.001, p*_t_* < 0.001, (**a**)), NEFA (p_CFP×*t*_ = 0.016, p_CFP_ = 0.021, p*_t_* < 0.001, (**b**)), BHB (p_CFP×*t*_ < 0.001, p_GLY×*t*_ = 0.007, p_GLY_ = 0.011, (**c**)) and cholesterol (p_GLY×CFP×*t*_ = 0.023, p_GLY×*t*_ = 0.027, p*_t_* < 0.001, p_GLY_ = 0.032, (**d**)). For all other variables: *p* > 0.05 (GLY, CFP, *t*, GLY×*t*, CFP×*t*, GLY×CFP, GLY×CFP×*t*). BHB = β-hydroxybutyrate; NEFA = non-esterified fatty acids; *t* = experimental time.

Serum glucose levels decreased in all groups until week 7 with higher levels in the HC groups followed by an increase until week 15 (p_CFP×*t*_ < 0.001, Figure 1a, Appendix A, Table A2). NEFA levels showed the opposite pattern with an increase until week 7 and higher levels in the LC groups, while levels afterwards decreased in the GLY_LC_ and HC groups (p_CFP×*t*_ = 0.016, Figure 1b, Appendix A, Table A2). Serum concentrations of BHB remained stable in all groups until week 7. Afterwards, BHB levels increased in GLY_HC_, and decreased in the LC groups until week 15 (p_CFP×*t*_ < 0.001, p_GLY×*t*_ = 0.007, Figure 1c, Appendix A, Table A2). Serum cholesterol concentrations showed a temporary decrease in all groups in week 7, which was more pronounced in the GLY groups, and increased again to initial levels in week 15. This, however, was not so pronounced for GLY_HC_ (p_GLY×CFP×*t*_ = 0.023, Figure 1d, Appendix A, Table A2). Total protein (p_GLY×*t*_ = 0.018, p_CFP×*t*_ = 0.003) and triglycerides (p_GLY×*t*_ = 0.039, p_CFP×*t*_ = 0.014) levels varied in all groups during the course of the experiment, while albumin concentrations (p_CFP_ < 0.001, p*_t_* < 0.001) and GLDH activity (p_CFP_ < 0.025) were mostly higher in the HC groups (Table 4). Conversely, total bilirubin levels were higher in the LC groups increasing over time, while they decreased in the HC groups (p_CFP×*t*_ = 0.001). Uric acid levels (p*_t_* < 0.001), as well as alkaline phosphatase (p*_t_* < 0.001) activity, decreased until week 7 and increased afterwards in almost all the experimental groups irrespective of any treatment. Urea concentrations (p*_t_* < 0.001) increased until week 7 followed by relatively stable values until week 15. AST activity varied over time in all groups (p_GLY×*t*_ = 0.046), whereas GGT activity remained unaffected by any treatment (Table 4).

#### 3.2.2. Hematology and Antioxidative Status

The HC groups displayed higher erythrocyte counts than the LC groups with the highest levels in week 7 (p_CFP_ = 0.021, p*_t_* < 0.001, Table 5). The red blood profile was not affected by GLY, whereas CFP and time had an influence on most analyzed parameters (Table 5). Compared with the LC groups, mean corpuscular volume (p_CFP×*t*_ < 0.001) and hematocrit (p_CFP×*t*_ = 0.007) levels were increased in the HC groups over the experimental time. Mean corpuscular hemoglobin concentrations (p_CFP×*t*_ = 0.002) in the HC groups were lower at week 7 and higher at week 15 compared with the LC groups (Table 5). In all groups, hemoglobin levels increased until week 15 with significantly higher levels in the HC groups than in the LC groups, whereas, in week 7, the CON groups displayed slightly higher hemoglobin than the GLY groups (p_GLY×*t*_ < 0.01, p_CFP×*t*_ < 0.001, Table 5). Mean corpuscular hemoglobin concentration levels marginally decreased until week 7 with the lowest level in GLY_HC_, while an increase was observed for all groups between week 7 and week 15 (p_GLY×*t*_ < 0.01, p_CFP×*t*_ < 0.01, Table 5). Platelet counts were highest in week 7 and red cell distribution width decreased significantly in all groups over the experimental time (p*_t_* < 0.001, Table 5). Both parameters remained unaffected by any treatment. In the HC groups, platelet distribution width (p_CFP×*t*_ < 0.01) and mean platelet volume (p_CFP×*t*_ = 0.029) showed higher levels over the course of 15 weeks compared with the LC groups (Table 5).

**Figure 2:**
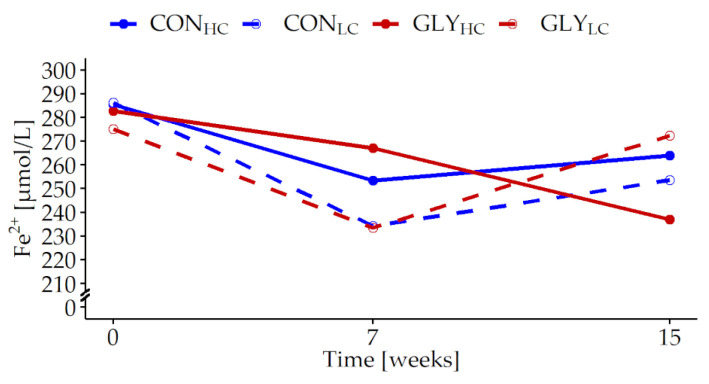
Ferric reducing ability of plasma (FRAP) of German Holstein bulls fed with glyphosate-contaminated or control rations and varying concentrate feed proportions for 15 weeks. Bulls were fed with GLY-contaminated (GLY groups, red lines) or control (CON groups, blue lines) rations and with low (LC, 1kg/animal/d, dashed lines) or high (HC, 2.5–5 kg/animal/d, solid lines) concentrate feed proportions (CFP) for 15 weeks (CON_HC_, *n* = 12; CON_LC_, *n* = 12; GLY_HC_, *n* = 11; GLY_LC_, *n* = 12). Values are depicted as LS means. Parameters were analyzed with values from week 0 as covariate. Significant *p*-values: p_GLY×CFP×*t*_ = 0.008, p_CFP×*t*_ = 0.001, p*_t_* < 0.001; *t* = experimental time.

With regard to the blood’s antioxidative status during the trial, the activities of the antioxidative enzymes GPx and SOD in erythrocytes were not affected by dietary treatment after 15 weeks of GLY exposure. GPx activity was 291.37 ± 27.30 (LS means ± standard error; CON_HC_), 277.91 ± 28.51 (CON_LC_), 261.22 ± 28.51 (GLY_HC_) and 279.24 ± 27.30 (GLY_LC_) mU/mg hemoglobin in the respective groups, while SOD reached activities of 3861.81 ± 309.05 (CON_HC_), 4286 ± 322.80 (CON_LC_), 3701.25 ± 322.80 (GLY_HC_), 3461.09 ± 309.05 U/mg hemoglobin (GLY_LC_). The ferric-reducing ability of serum decreased in all groups until week 7, with higher values in the HC groups than in the LC groups. Between week 7 and week 15, the ferric reducing ability of serum further decreased in GLY_HC_, while in the remaining groups the values increased (p_GLY×CFP×*t*_ < 0.01, Figure 2, Appendix A, Table A3).

**Figure 3:**
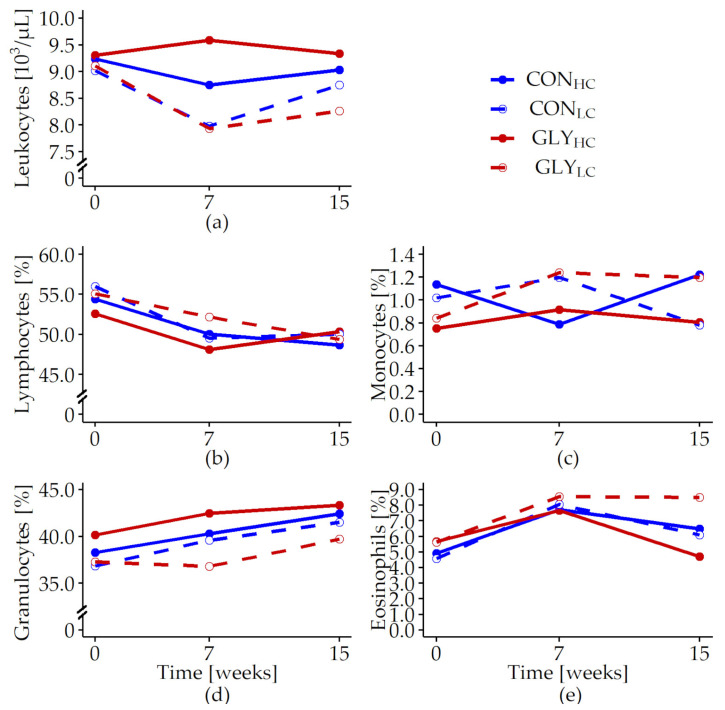
Influences of glyphosate residues and different concentrate feed proportions on cell populations in German Holstein bulls. Shown are the development of leukocytes (**a**), lymphocytes (**b**), monocytes (**c**), granulocytes (**d**) and eosinophils (**e**). German Holstein bulls were fed with glyphosate-contaminated (GLY groups, red lines) or control (CON groups, blue lines) rations and with low (LC, 1kg/animal/d, dashed lines) or high (HC, 2.5–5 kg/animal/d, solid lines) concentrate feed proportions (CFP) for 15 weeks (CON_HC_, *n* = 12; CON_LC_, *n* = 12; GLY_HC_, *n* = 11; GLY_LC_, *n* = 12). Values are represented as LS means. Parameters were analyzed with values from week 0 as covariate, if the first sampling point had a significant influence. Significant *p*-values for leukocytes (p_CFP_ < 0.001, p*_t_* = 0.023, (**a**)), lymphocytes (p*_t_* < 0.001, (**b**)) and granulocytes (p_CFP_ = 0.037, p*_t_* = 0.042, (**d**)). For all other variables: *p* > 0.05 (GLY, CFP, *t*, GLY×*t*, CFP×*t*, GLY×CFP, GLY×CFP×*t*). *t* = experimental time.

Within the white blood profile, cell counts of total leukocytes were higher and remained relatively stable over the time in the HC groups compared with the LC groups, which displayed minimum cell counts in week 7 (p_CFP_ < 0.001, p*_t_* = 0.023, Figure 3a, Appendix A, Table A4). Proportions of lymphocytes varied over time (p*_t_* < 0.001, Figure 3b, Appendix A, Table A4) and monocytes remained unaffected by any treatment or experimental time (Figure 3c, Table A4). Granulocytes were higher in the HC groups and increased constantly over time, which was also observed in CON_LC_ (p_CFP_ = 0.037, p*_t_* = 0.042, Figure 3d, Appendix A, Table A4). In contrast, the proportions of eosinophils remained unaffected by any treatment or time (Figure 3e, Appendix A, Table A4). 

#### 3.2.3. DNA Damage Indicators in Leukocytes

After 15 weeks of oral GLY exposure, a comet assay of bovine leukocytes revealed an interactive influence of CFP and GLY on the proportion of tail DNA (p_GLY×C_ = 0.003, p_GLY_ = 0.034) and Olive tail moment (p_GLY×CFP_ = 0.003, p_GLY_ = 0.014) with the highest levels in CON_HC_ and the lowest levels in GLY_HC_ (Table 6). 

#### 3.2.4. Functional Properties of Immune Cells

**Figure 4:**
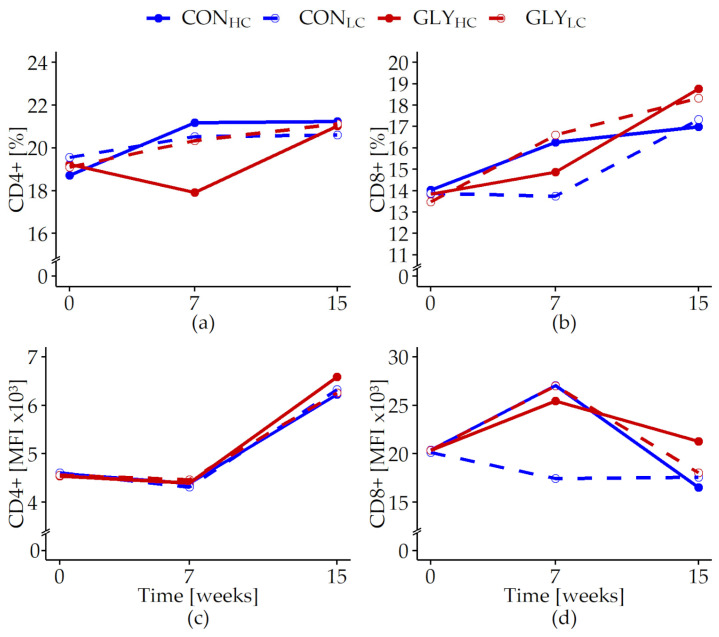
Relative proportions and mean fluorescence intensity (MFI) of CD4+ and CD8+ cells of total peripheral blood mononuclear cells in fattening German Holstein bulls fed with glyphosate residues and different concentrate feed proportions in the rations for 15 weeks. Shown are the proportions of CD4+ (**a**) and CD8+ (**b**) cells as well as respective MFI (CD4+: (**c**); CD8+: (**d**)). Bulls were fed with glyphosate-contaminated (GLY groups, red lines) or control (CON groups, blue lines) rations and with low (LC, 1kg/animal/d, dashed lines) or high (HC, 2.5–5 kg/animal/d, solid lines) concentrate feed proportions (CFP) for 15 weeks (CON_HC_, *n* = 12; CON_LC_, *n* = 12; GLY_HC_, *n* = 11; GLY_LC_, *n* = 12). Values are represented as LS means. Parameters were analyzed with values from week 0 as covariate, if the first sampling point had a significant influence. Significant *p*-values for CD4+ (%) (p_GLY×*t*_ = 0.048, p*_t_* < 0.001, (**a**)), CD8+ (%) (p_GLY×CFP×*t*_ = 0.003, p*_t_* < 0.001, (**b**)), CD4+ (MFI) (p_GLY×CFP×*t*_ = 0.049, p*_t_* < 0.001, (**c**)) and CD8+ (MFI) (p_GLY×CFP×*t*_ < 0.001, p_CFP×*t*_ = 0.044, p_GLY×*t*_ = 0.009, p_CFP_ = 0.005, p_GLY_ < 0.001, p*_t_* < 0.001, (**d**)). For all other variables: *p* > 0.05 (GLY, CFP, GLY×*t*, CFP×*t*, GLY×CFP, GLY×CFP×*t*). *t* = experimental time.

In addition to the analyses of blood cell populations and in order to examine the putative consequences of measured DNA damage on leukocytes’ properties, analyses of subpopulations of lymphocytes expressing CD4 molecule representing T helper cells as well as CD8 expressing cells representing cytotoxic T cells were conducted (Figure 4, Appendix A, Table A5).

Proportions of CD4+ cells displayed opposite patterns between experimental groups in week 7 with an increase in CON_HC_ and a decrease in GLY_HC_. In week 15, however, levels were similar in both groups (p_GLY×*t*_ < 0.043, Figure 4a, Appendix A, Table A5).

Proportions of CD8+ cells increased inconstantly in CON_LC_, GLY_HC_, GLY_LC_ until week 15 with the highest values in GLY_HC_. In CON_LC_, the proportions of CD8+ cells showed a slight decrease in week 7 followed by an increase until week 15 (p_GLY×*t*_ = 0.003, Figure 4b, Appendix A, Table A5).

In all experimental groups, MFI of CD4+ cells were highest in week 15 after a slight decrease between week 0 and week 7 (p_GLY×CFP×*t*_ = 0.049, Figure 4c, Appendix A, Table A5).

MFI of CD8+ cells increased in CON_HC_, GLY_HC_, GLY_LC_ until week 7 followed by lower levels in week 15, while MFI of CD8+ cells in CON_LC_ showed a slight decrease until week 7 followed by a slight increase in week 15 (p_GLY×CFP×*t*_ < 0.001, Figure 4d, Appendix A, Table A5).

An evaluation of Concanavalin A stimulated proliferation of PBMC by alamar blue assay showed no significant influences of dietary treatment after 15 weeks on the stimulation index, which amounted to 3.47 ± 0.22 (mean ± standard error; CON_HC_), 3.39 ± 0.11 (CON_LC_), 3.18 ± 0.16 (GLY_HC_) and 3.69 ± 0.24 (GLY_LC_), respectively.

**Figure 5:**
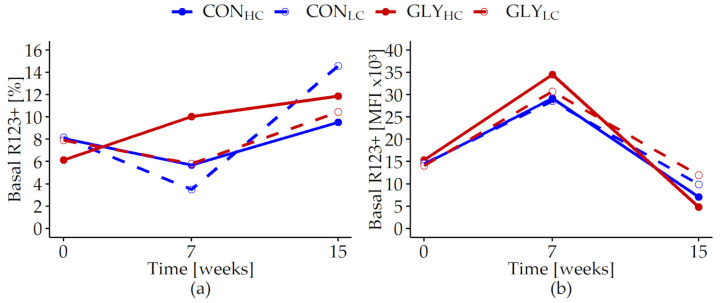
Reactive oxygen species production in polymorphonuclear leukocytes of fattening German Holstein bulls fed rations with or without glyphosate residues and different concentrate feed proportions for 15 weeks. Presented are the proportions (%, (**a**)) and the mean fluorescence intensities (MFI, (**b**)) of unstimulated (basal) rhodamine positive cells (R123+). Bulls were fed with glyphosate-contaminated (GLY groups, red lines) or control (CON groups, blue lines) rations and with low (LC, 1kg/animal/d, dashed lines) or high (HC, 2.5–5 kg/animal/d, solid lines) concentrate feed proportions (CFP) for 15 weeks (CON_HC_, *n* = 12; CON_LC_, *n* = 12; GLY_HC_, *n* = 11; GLY_LC_, *n* = 12). Values are presented as LS means. If week 0 had a significant influence (*p* < 0.05) in a parameter, the respective value was used as covariate. Significant *p*-values for basal R123+ (%) (p*_t_* < 0.001, (**a**)) and MFI (p_GLY×CFP×*t*_ = 0.002, p_CFP×*t*_ < 0.001, p_GLY_ = 0.033, p*_t_* < 0.001, (**b**)). For all other variables: *p* > 0.05 (GLY, CFP, GLY×*t*, CFP×*t*, GLY×CFP, GLY×CFP×*t*). *t* = experimental time.

The measurements of intracellular ROS production in PMN showed inconsistently varying proportions of basal ROS forming cells over time, unaffected by any treatment (p*_t_* < 0.001, Figure 5a, Appendix A, Table A6).

The MFI of the basal ROS producing PMN increased until week 7 with the highest MFI in GLY_HC_, followed by a decrease until week 15 to levels below those observed at the beginning of the trial for all groups (p_GLY×CFP×*t*_ = 0.002, Figure 5b, Appendix A, Table A6). Stimulated PMN proportions as well as quantities of ROS in these cells showed lower levels in week 7 than in week 0, while an increase back to levels observed at the beginning of the trial was observed until week 15 (p*_t_* < 0.001, Table 7). In week 15, proportions of phagocytic PMN were elevated in the HC groups compared with levels in week 0, whereas levels in the LC groups were lower than in week 0 (p_CFP×*t*_ = 0.004). During the course of the experiment, the capacity of the phagocytic cells slightly decreased in GLY_HC_ and CON_LC_ within the first 7 weeks. Afterwards, an elevation in the capacity of phagocytic cells was observed in all experimental groups with the strongest pronunciation in the HC groups (p_CFP×*t*_ = 0.04, Table 7).

After 15 weeks of oral GLY exposure, the proportions of early and late apoptotic blood cells displayed no significant effects related to treatments (Table 8). 

### 3.3. Gene Expression Analysis

In blood leukocytes, mRNA abundances of *BCL2* (p_GLY_ = 0.042), *HSPA1A* (p_GLY_ = 0.043), *PARP1* (p_GLY_ = 0.008), *TP53* (p_GLY_ = 0.002) and *XRCC5* (p_GLY_ = 0.024) were higher in the GLY groups, while *CDKN1A* (p_CFP_ = 0.01), *NRF2* (p_CFP_ = 0.002) and *SOD2* (p_CFP_ < 0.001) displayed increased mRNA abundances in the HC groups. *BRCA1*, *CASP3, GADD45A*, *OGG1* and *RAD51* mRNA abundances were not significantly influenced by any treatment (Table 9).

## 4. Discussion

**Figure A1:**
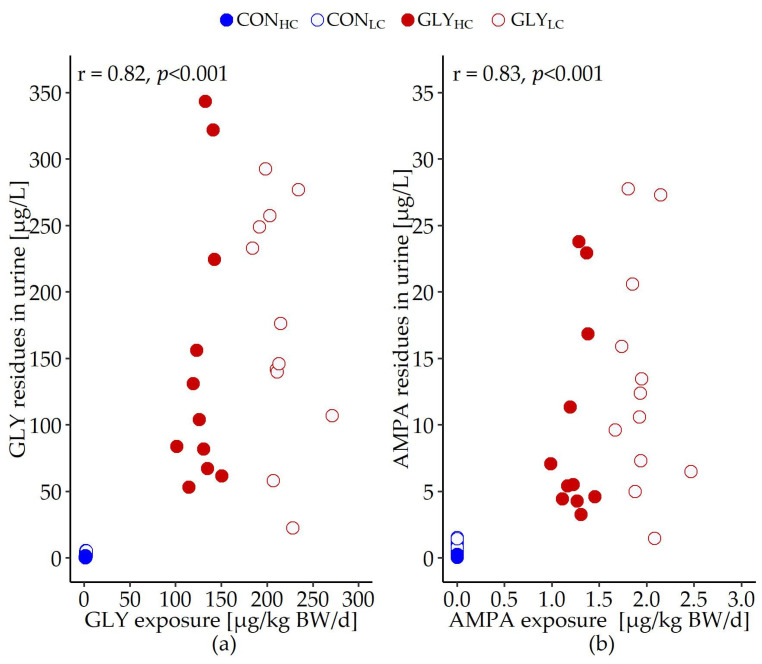
Spearman’s rank correlation of average glyphosate (GLY, (**a**)) and AMPA exposure (**b**) with respective concentrations in urine before slaughtering of fattening bulls after GLY exposure for several weeks. Fattening bulls (*n* = 47) were fed with (GLY groups) or without GLY residues (CON groups) in the ration and low (LC) or high (HC) concentrate feed proportions.

As a frequently used active substance in non-selective herbicides, GLY and its potential toxic effects on animal health resulting from contaminated feedstuffs are a focus of scientific interest [6,7,9,20,47]. Since GLY residues can often be detected in livestock feed [5], their effects on the health and performance of ruminants are of central concern. Thus, this study aimed to analyze whether GLY residues in feedstuffs resulting from permitted herbicide usage and representing formerly real-life GLY exposure conditions in Germany influence the health of growing bulls. The average daily GLY exposure (76 mg/d) in the GLY groups was comparable to the average exposure in a similar trial with dairy cows (79 mg/d, [7,8]), whereas it was 11× higher than the maximum background exposure of dairy cows at the same Research Institute under feeding conditions with imported soybean meal [5]. In the present investigation, the average GLY exposure translated to individual BW was even 53× higher (GLY_LC_, 213.7 µg/kg BW/d) than the observed maximum background exposure of dairy cows [5]. Although DMI was higher in HC groups, GLY_LC_ showed the highest average GLY exposure. This can be explained by the fact that straw as part of the roughage (21% straw, 79% maize silage) had the highest GLY concentrations among the feedstuffs used in this study [8]. Since the LC groups received lower CFP (1 kg/animal/d), intakes of roughages were higher leading to an increased intake of GLY-contaminated straw and consequently also increased GLY exposure. Likewise, mean GLY concentrations in urine before slaughtering were higher in this study (148.1 µg/L in GLY_HC_, 188.0 µg/L in GLY_LC_) than concentrations measured in dairy cows exposed to background contaminations of GLY (lower than the LOQ—75.1 µg/L, [5]). Significant correlations between mean GLY and AMPA exposures and corresponding concentrations in urine before slaughtering suggested that parts of ingested GLY and AMPA were systemically available and finally eliminated via urine (Appendix A, Figure A1, [5]). While AMPA exposure in the present study amounted to approximately 1% of the total exposure (GLY + AMPA) in GLY groups (0.99% GLY_HC_, 0.86% GLY_LC_), urinary concentrations of AMPA reached 6.33% (GLY_LC_) and 7.01% (GLY_HC_) of the total concentrations (GLY + AMPA), indicating putative ruminal GLY metabolization to AMPA [5]. Since a GLY formulation was applied to feedstuff in the present study and therefore cattle were also exposed to other formulation ingredients besides GLY, AMPA concentrations could additionally result from the degradation of other formulation detergents as reviewed [48]. 

In the present study, DMI, average daily gain as well as feed conversion were not affected by GLY, which is in accordance with [9] who fed weaning pigs with 0, 10, 20 and 40 mg GLY/kg diet for 35 days, [21] where no effects on performance and carcass characteristics were observed when feeding GLY-tolerant corn to finishing feedlot steers and [8] where dairy cows were fed a diet that was prepared from the same feedstuffs used in the present study. 

**Figure A2:**
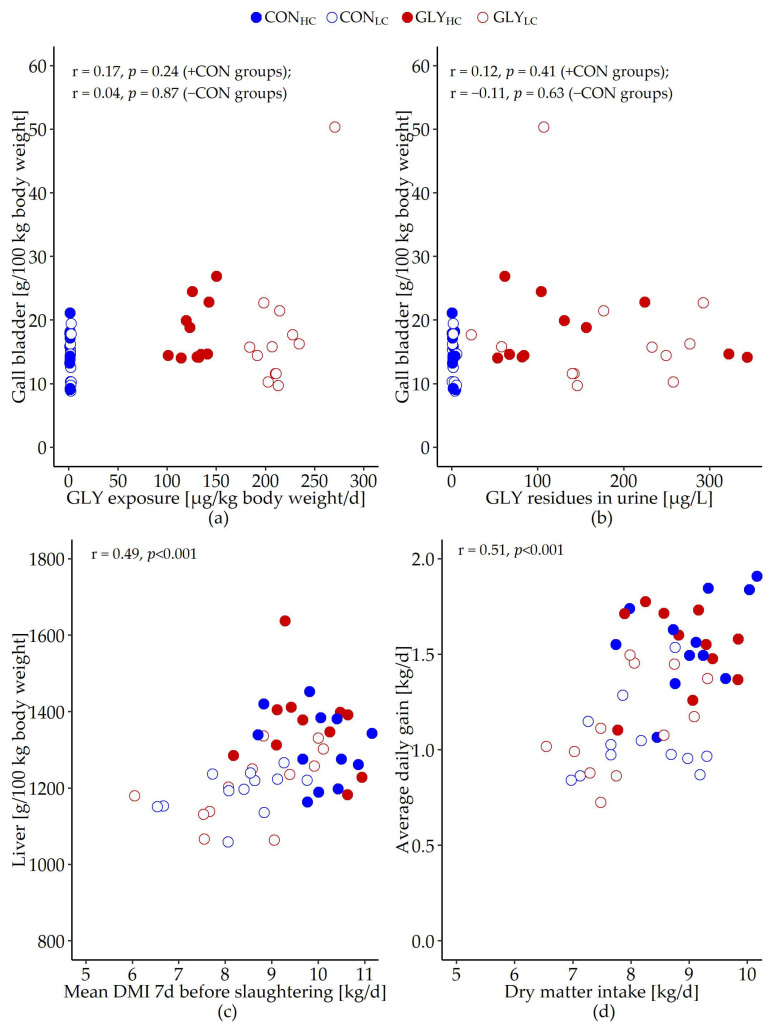
Spearman’s rank correlation of organ weights relative to body weight and performance with glyphosate (GLY) exposure and urinary residue concentration of fattening bulls after GLY exposure. Scatter plots show relative gall bladder weights against GLY exposure (**a**), urinary GLY concentration (**b**) and relative liver weights against mean dry matter intake (DMI) 7 days before slaughtering (**c**), as well as mean DMI and average daily gain (**d**). Fattening bulls (*n* = 47) were fed with (GLY groups) or without GLY residues (CON groups) in the ration and low (LC) or high (HC) concentrate feed proportions.

The relative weights of almost all assessed organs remained unaffected by GLY, whereas higher CFP usually led to increased organ weights relative to BW. Similarly, in weaning pigs the influence of GLY on absolute and relative organ weights (liver, heart, spleen, lung and kidney) was not observed after oral treatment with 10, 20 and 40 mg GLY/kg diet for 35 days [9]. In contrast, significantly increased weights of kidney, liver and testis in male guinea pigs were reported after oral treatment with 67, 103 and 202 mg GLY/kg BW for 60 days [49], which is increased by orders of magnitude in comparison with GLY exposure with relation to practical usage. The weights of gall bladders were significantly affected by GLY, with the present study displaying higher weights in GLY groups. In general, one possible reason for elevated gall bladder weights in cattle is the increased thickness of the wall due to cholestasis or inflammation [50]. However, neither GGT in serum indicating cholestasis nor inflammatory markers such as total leukocytes or albumin were significantly influenced after 15 weeks of GLY exposure. Finally, no significant correlations between GLY exposure (Figure A2a) or urinary residue concentration (Figure A2b) and gall bladder weight were detected, while correlations between DMI and relative liver weight (Figure A2c)) or average daily gain (Figure A2d) were clearly visible, significant and CFP-dependent. In summary and as hypothesized, GLY residues in feedstuffs resulting from common, formerly legal, agricultural practice did not compromise the performance-related parameters as well as slaughter characteristics in growing bulls.

As already mentioned, GLY neither affected the enzyme activities of alkaline phosphatase, GLDH and GGT, nor the parameters such as albumin and total bilirubin in the present study, while CFP and/or time had significant impacts. These findings are in contrast to [13] and [14], who reported increased activities of GGT in albino Swiss mice or decreased albumin, blood protein and increased total bilirubin levels in rats upon oral GLY exposure. Again, GLY exposures were largely higher in the aforementioned studies when compared with exposure resulting from common agricultural practice presented in the current study. In comparison, energy metabolism related to the blood parameters, glucose, NEFA, triglycerides and BHB, showed significant CFP and time effects in the present study, which was also reported for dairy cows under similar feeding conditions [8]. Although GLY influences were significant for AST, BHB, cholesterol, triglycerides and total serum protein in the present study, the aforementioned parameters were within the physiological ranges for cattle [51] and comparable to levels measured in animals investigated in another trial at the Institute of Animal Nutrition in Braunschweig [52]. This indicates that the observed effects did in fact not compromise animal health and might be incidental findings. Discrepancies between studies on cattle and the studies on laboratory animals discussed above could be based on much higher GLY exposure in relation to BW.

Next to clinical-chemical traits, the hematologic profile of the fattening bulls was of interest, since the literature indicated GLY-induced hematological alterations in mice and rats [13,14]. In the present study, various red blood profile-associated parameters were affected by CFP and time, which is in accordance with the results in dairy cows [7]. Moreover, hemoglobin and mean corpuscular hemoglobin concentration were significantly affected by GLY and time next to an effect of CFP over time. The levels of hemoglobin, in fact, became stronger over time in groups fed with high CFP, resulting in marked differences between the HC and LC groups after 15 weeks. However, temporarily decreased (GLY_HC_ compared to CON_HC_) and simultaneously increased (CON_LC_ compared to GLY_LC_) hemoglobin levels at week 7 might have caused the mentioned significant GLY effect. Again, GLY effects appeared as components of interactions in an inconsistent manner indicating putative statistical artifacts.

Decreases in red blood cell counts, confirmed by a reduction of hemoglobin levels, were postulated to be caused by Roundup-induced ROS production [14]. Having a closer look at the antioxidative system, no significant changes of SOD and GPx activities in the plasma of rats exposed to GLY (10 mg/kg BW [53]) were reported, in accordance with the results of the present study. However, a decreased ferric reducing ability of plasma (FRAP) was observed [53]. In fattening bulls, although a constant decrease in FRAP was observed in GLY_HC_, while not being significantly different to CON_HC_ at week 15 in a post hoc test (Tukey), GLY_LC_ showed the highest level of all groups at week 15. This and less consistent GLY effects compared with CFP effects hint at the observed effect being an incidental finding rather than a physiologically relevant observation.

However, increased ROS levels might lead to oxidative stress and, consequently, if not balanced by the antioxidative defense system, could result in DNA damage, for example in bovine leukocytes [16]. Although an induction of single and double strand breaks by GLY was shown in human PBMC in vitro [17], the results of the comet assay in the present study indicated higher values for Olive tail moment and DNA content in the comet tail in CON_HC_ than in GLY_HC_. This is in agreement with the observation that GLY did not lead to DNA damage in bovine leukocytes in vivo [7]. If DNA damage had occurred to a level that cannot be balanced by the cells, it would have consequently affected further parameters, e.g., total leukocytes and individual cell populations in the blood as well as their functional properties. Regarding the white blood profile, no adverse effects of GLY were observed in the present study, whereas CFP affected total leukocyte and granulocyte proportions. These results were in accordance with the results in dairy cows under conditions relevant to practical application [7] and indicate that the general blood cell composition was not negatively influenced by GLY. Although total leukocytes in general and lymphocytes in particular were not influenced by GLY, proportions of CD4+ and CD8+ lymphocytes, as subtypes of T-lymphocytes, displayed inconsistent but significant changes over time, differing between all groups. In general, the mean proportions of CD4+ (20.1%) and CD8+ (15.6%) were comparable to values measured in growing bulls at the Institute of Animal Nutrition in Braunschweig (CD4+ 21.9%; CD8+ 14.6%, [52]). Additionally, the proportions of CD4+ and CD8+ in GLY-exposed dairy cows were not affected by GLY [7], indicating that the observed findings do likely not reflect the negative impacts on T-lymphocytes.

In contrast to T-lymphocytes, which are involved in the cell-mediated immune response, PMN, mainly neutrophils, are part of the innate immune system and responsible for killing invading pathogens by producing large amounts of ROS [15,54]. Additionally, ROS are physiologically generated at low levels as cellular messengers in multiple signaling pathways relating to cell growth, migration or T-cell activation [15,16].

In the present study, the intracellular amount of ROS in unstimulated PMN showed highest levels at week 7 in all groups, while after 15 weeks of treatment, ROS production of PMN was lower than at the beginning of the trial. Particularly elevated PMN levels at week 7 in GLY_HC_ and comparably more pronounced differences between LC groups and HC groups after 15 weeks led to a significant interactive influence of GLY, CFP and time. A similar time-related pattern of ROS production at its highest levels after several weeks of feeding experimental diets followed by a strong decrease was also observed in dairy cows under similar feeding conditions [7] and might be an adaptation to applied changes in the diet. However, the proportions of basal and stimulated PMN were neither affected by CFP, GLY nor a combination of both, and only changed over time.

**Figure A3:**
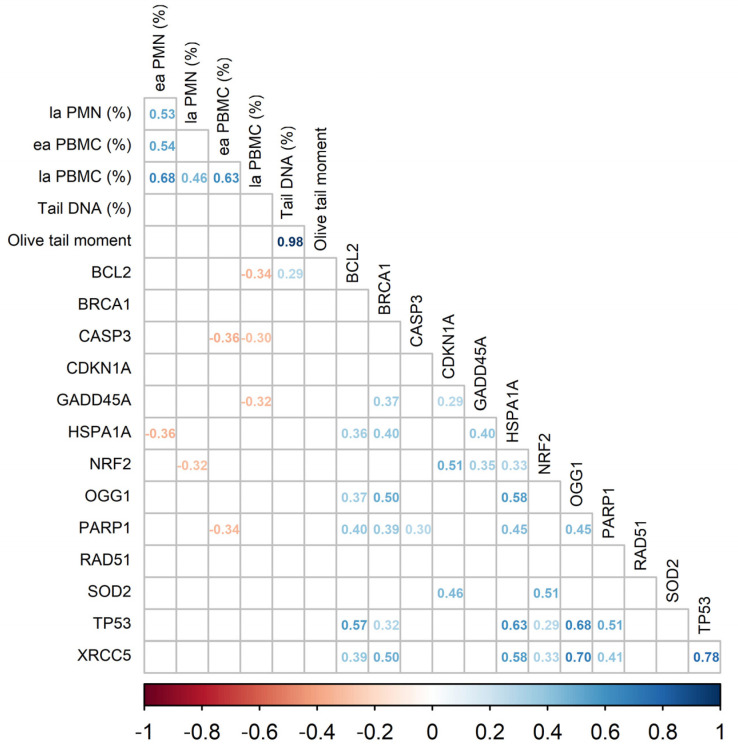
Significant Spearman’s rank correlation coefficients of relative expression levels of genes of interest in leukocytes and comet assay data (tail DNA (%), Olive tail moment) as well as early and late apoptotic (ea, la) polymorphonuclear leukocytes (PMN) and peripheral blood mononuclear cells (PBMC) after 15 weeks. Fattening bulls (*n* = 47) were fed with (GLY groups) or without GLY residues (CON groups) in the ration and low (LC) or high (HC) concentrate feed proportions.

Among ROS-related genes in bovine leukocytes, mRNA abundance levels of *SOD2* and *Nrf2*, encoding proteins involved in ROS sensing [16,55] were increased in the blood leukocytes of HC groups, indicating an increased cell metabolism as a consequence of enhanced energy availability [28]. Although ROS can lead to DNA damage, the results regarding mRNA abundances of ROS sensing genes and elevated proportions of DNA in comet tail indicating DNA damage were not correlating (Figure A3). Considering the observed elevated mRNA abundance of DNA repair-related genes (*HSPA1A*, *XRCC5*, *PARP1* and *TP53*) in the GLY groups on the one hand and on the other hand the decreased mRNA abundances in the CON groups, different levels of activity in DNA repair mechanisms might be an explanation for the different DNA proportions in the comet tail. Another putative reason for differences in the comet assay might be a coincidentally different presence of cells in the S-phase of the cell cycle between experimental groups, since replication forks in S-phase could lead to comet tails using the alkaline method [56]. The absence of observed GLY effects was in contrast to [57], where human Hep-2 cells showed increased DNA damage in a comet assay after GLY exposure in vitro. Together with p53 (protein encoded by *TP53*), PARP1 is involved in the response to DNA single strand breaks. The p53 protein is an essential transcription factor for genes involved in DNA repair, cell cycle regulation (e.g., *GADD45A*, *CDKNA1*) [58], apoptosis (e.g., *BCL2*) [59] and further mechanisms depending on stress signals [58,60]. In human PBMC treated with 100 µM GLY comparable to levels detected in acutely intoxicated humans, levels of *TP53* were significantly decreased, while levels of *P21* and *BCL2* were increased [61]. Although mRNA abundances of apoptosis-related *BCL2*, DNA repair-associated *HSPA1A*, *XRCC5*, *PARP1* and *TP53* were slightly increased in GLY groups in this study, the results were only weakly correlated with the results of the comet assay (*BCL2*, R = 0.29, Figure A3) and apoptotic cells (*BCL2*: R = −0.34, *HSPA1A*: R = −0.36, *PARP1*: R = −0.34, Figure A3). However, the proportions of early and late apoptotic PMN and PBMC were neither influenced by GLY residues nor by different CFP. These results were in contrast to [19], who reported an increase in apoptotic human PBMC treated with 0.5 and 5 mM GLY in vitro corresponding to GLY levels detected in the blood of humans poisoned with GLY formulations. The absence of observable GLY effects on phagocytosis and apoptosis related parameters in this study are in line with the results in German Holstein dairy cows exposed to GLY concentrations observable in practical application [7]. Although signaling pathways are strictly regulated networks, the biological consequences of putative genotoxic effects of GLY in leukocytes could not be supported by analyses of apoptotic cells or of the functional properties of immune cells and need further clarification.

As has already been discussed [6,7,8], discrepancies between the results of the present study and the studies discussed above might be related to lower GLY concentrations and the different surfactants of the tested GLY formulation in comparison with previous studies. Nevertheless, the applied GLY concentration in the present study represents conditions that could formerly legally occur in agricultural practice, while the discussed studies typically used in vitro cell systems or laboratory adapted model organisms in combination with elevated GLY concentrations that do not occur in vivo under conditions relevant to practical application. Since only three time points within the 15 weeks were evaluated, the differences between groups at one time point should be evaluated individually in addition to analyses over the course of trial. Although our data showed GLY effects for selected traits, it needs to be pointed out that these findings were mostly characterized by high individual variation and were comparable to ranges already observed in other studies. Furthermore, the observed interactive effects between GLY with CFP and/or time occurred in an inconsistent manner and are most likely not reproducible. As the magnitude of these GLY effects was low in comparison with the marked and consistent CFP effects, the toxicological relevance of the observed GLY effects remains doubtful. Finally, all animals remained clinically inconspicuous throughout the trial. Taking together the results of the present study, GLY residues in feedstuffs resulting from common, formerly legal, herbicide usage in Germany did not compromise the general health of growing Holstein bulls throughout fattening phase. However, a possibly existing relevance of the GLY effects observed in this study should be addressed in further studies including lifetime exposure studies.

## 5. Conclusions

The glyphosate exposure in the present study which represented a real-life scenario under formerly legal conditions in Germany and resulted in an average daily GLY exposure of 171 µg/kg BW/d in GLY groups did not adversely influence performance or organ weights, as well as the blood parameters, functional properties of blood cells or DNA integrity-related parameters in fattening bulls. In contrast to this, varying proportions of concentrate feed and the time that the fattening diets were fed influenced various performance-related and physiological parameters. While spuriously statistically significant effects occurred in relation to GLY exposure, these effects might be incidental since they were not consistent between the parameters and sample sizes as well as the number of samplings being limited. Most importantly, all animals remained clinically inconspicuous and the levels of most parameters putatively affected by GLY were within physiological ranges.

## Figures and Tables

**Table 1:** Components and chemical composition of concentrates and roughages in experimental diets.

	Concentrates				Roughage		
	Experimental Group						
	GLY_HC_ *n* = 11	CON_HC_ *n* = 12	GLY_LC_ *n* = 12	CON_LC_ *n* = 12	Maize Silage	Straw CON	Straw GLY
Components [% dry matter]							
Peas		35.0		65.0			
Peas GLY-treated	35.0		65.0				
Wheat		29.0		10.0			
Wheat GLY-treated	29.0		10.0				
Corn	29.2	29.2					
Soybean oil	1.0	1.0	1.0	1.0			
Urea	1.8	1.8	13.0	13.0			
Calcium carbonate	1.0	1.0					
Vitamin/Mineral Premix ^$^	3.0	3.0	11.0	11.0			
Chemical composition							
Dry matter (DM) [%]	87	87	87	89	34	88	86
Nutrients [g/kg DM]							
Crude ash	61	60	127	131	37	60	57
Crude protein	203	202	554	560	70	34	32
Ether extract	41	42	26	24	35	15	14
Crude fibre	40	36	44	45	190	443	427
aNDF_om_^‡^	97	95	85	77	379	820	821
ADF_om_^§^	48	46	56	57	219	505	500
Starch	582	595	409	402	367		
Sugar	44	43	41	42			
Energy [MJ ME/kg DM]	12.8	12.8	10.4	10.4	11.5	6.4	6.4
Herbicide agent residues [mg/kg DM]							
Glyphosate	0.41	0.02	0.31	0.01	0.00 ^#^	0.57 ^#^	61.81 ^#^
AMPA	0.01	0.00	0.00	0.00	0.00 ^#^	0.00 ^#^	0.59 ^#^

Values are presented as means. Chemical compositions were calculated based on the standard methods of VDLUFA for individual feedstuffs also used in [8] and according to [8] (^#^); AMPA = aminomethylphosphonic acid (degradation product of glyphosate); CFP = concentrate feed proportion; CON = control; DM = dry matter; GLY = glyphosate; HC = high CFP (2.5–5 kg/animal/d); LC = low CFP (1 kg/animal/d); ME = metabolizable energy; ^$^ provided per kg concentrate feed (according to manufacturer specification); ^‡^ aNDFom, neutral detergent fibre without ash, amylase treated, ^§^ ADFom, acid detergent fibre without ash.

**Table 2:** Information about gene-specific primers used for qRT-PCR.

Gene Symbol	NCBI Accession Number	Sequences [fw/rev] 5′-3′	Concentration [nmol] fw/rev	Amplicon Size [bp]	Efficiency [%]	Reference
Reference genes						
*RPS9*	NM_001101152.2	F-GGCGTCTGTTCGAAGGTAATG R-GGGATGTTCACCACCTGCTT	333/333	229	101.8	this study
*UCHL*	NM_174481	F-CAAAGACAACTTGCTGAGGAACC R-ACTGCTTGTGTTCTGCTAAAGTC	333/333	208	95.9	[35]
*UXT*	NM_001037471.2	F-CGCTACGAGGCTTTCATCTCT R-CGAGTGGTTAGCTTCCTGGAGT	333/333	141	89.6	[6]
Genes of interest						
*BCL2*	NM_001166486.1	F-ACGGAGGCTGGGACGCCTTT R-AGGGTGATGCAAGCGCCCAC	250/333	121	96.8	[35]
*BRCA1*	NM_178573.1	F-CCAAAGCGAGCAAGAGAATCC R-CACTCTAGTTGATCTGTGGGC	333/333	101	94.8	this study
*CASP3*	NM_001077840.1	F-CAGCGTCGTAGCTGAACGTA R-AGGCCATGCCAGTATTTTCG	500/500	221	102	[35]
*CDKN1A*	NM_001098958.2	F-CCACTCCAAACGCAGACTGA R-GCACAAACTGAAGGCCCAAG	333/333	131	84.6	this study
*GADD45A*	NM_001034247.1	F-GAGCAAAAGACCGAAAGGATGG R-CAGGCACAGCACCACGTTAT	333/333	147	96.6	this study
*HSPA1A*	NM_203322.3	F-AGGACTTCGACAACAGGCTG R-TGCTGGACGACAAGGTTCTC	500/500	141	93.2	this study
*NRF2*	NM_001011678.2	F-AGCTCAGCATGATGGACTTGGA R-CAGCTCATGCTCCTTCTGTCG	333/333	152	91.1	[28]
*OGG1*	NM_001080285.2	F-ACCTTTGGACCTCGGCTCAT R-CCGTTCTTCTAGGATGGCTCG	333/333	165	94.4	this study
*PARP1*	NM_174751.2	F-CGGACAGATGTTTCAGGCAAAG R-TGGGGCTTATCGGGGTACA	250/250	179	97.1	[28]
*RAD51*	NM_001046179.2	F-GCCCTAGCGAATACCAAAGC R-ACCACATTGCTCTAGTCGGG	333/333	145	94.1	this study
*SOD*	NM_201527.2	F-CGTGACTTTGGTTCCTTTGCC R-GCGTCCCTGCTCCTTATTGA	250/250	108	95.2	[28]
*TP53*	NM_174201.2	F-TAGGAGCACTAAGCGAGCACT R-GTTCCCTTCCATCCAGAGCA	333/333	173	86.4	this study
*XRCC5*	NM_001102141.1	F-AGACCCTCTTCCCTCTGACG R-TTCACGCTTCCAACACAGGT	333/333	181	99.6	this study

*BCL2* = BCL2 apoptosis regulator; *BRCA1* = BRCA1 DNA repair associated; *CASP3* = Caspase 3; *CDKN1A* = cyclin dependent kinase inhibitor 1A; *GADD45A* = growth arrest and DNA damage inducible alpha; *HSPA1A* = heat shock protein family A (Hsp70) member 1A; *NRF2* = nuclear factor erythroid 2–related factor 2; *OGG1* = 8-oxoguanine DNA glycosylase; *PARP1* = poly(ADP-ribose) polymerase 1; *RAD51* = RAD51 recombinase; *SOD2* = superoxide dismutase 2; *TP53* = tumor protein p53; *XRCC5* = X-ray repair cross complementing 5.

**Table 3:** Performance characteristics during 15 weeks of the trial and selected parameters of slaughtering of fattening bulls (*n* = 47) fed with or without glyphosate-contaminated rations and varying concentrate feed proportions.

	Experimental Group					*p*-Value		
	CON_HC_ (*n* = 12)	CON_LC_ (*n* = 12)	GLY_HC_ (*n* = 11)	GLY_LC_ (*n* = 12)	PSEM	CFP	GLY	GLY× CFP
Final performance evaluation ^#^								
Dry matter intake (DMI) [kg/d]	9.0	8.1	8.9	7.9	0.090	**<0.001**	0.400	0.834
Water intake [l/d]	21.9	21.2	22.1	21.4	0.566	0.512	0.868	0.983
Energy intake [MJ/d]	105	85	103	83	0.957	**<0.001**	0.420	0.891
Average daily gain (ADG) [kg/d]	1.6	1.0	1.5	1.1	0.033	**<0.001**	0.669	0.321
Feed conversion [kg DMI/kg ADG]	6.0	8.3	6.0	7.6	0.189	**<0.001**	0.336	0.320
Energy utilization [MJ ME/kg ADG]	69.4	86.8	69.6	79.2	2.027	**0.001**	0.362	0.341
GLY exposure [µg/kg BW/d]	1.3	2.0	128.6	213.7	1.444	**<0.001**	**<0.001**	**<0.001**
AMPA exposure [µg/kg BW/d]	0.0	0.0	1.2	1.9	0.014	**<0.001**	**<0.001**	**<0.001**
Body weight before slaughtering [kg], selected organ weights [g/100 kg BW] and GLY/AMPA residues in urine [µg/L]								
Body weight	584.3	501.4	570.8	510.0	8.510	**<0.001**	0.886	0.520
GLY in urine	1.8	2.3	148.1	188.0	66.954	0.484	**<0.001**	0.503
AMPA in urine	0.3	0.6	10.0	13.2	5.657	0.296	**<0.001**	0.383
Gall blader	14.9	13.6	18.1	18.1	0.945	0.733	**0.046**	0.722
Heart	306.6	285.6	307.5	278.2	0.005	**0.014**	0.741	0.673
Kidneys	200.5	195.9	214.1	204.2	3.674	0.328	0.142	0.722
Liver	1306.9	1191.3	1361.7	1208.1	13.601	**<0.001**	0.196	0.490
Lung	557.6	510.6	539.5	498.3	9.786	**0.029**	0.441	0.884
Prostate gland	113.1	103.7	114.3	104.2	2.117	**0.027**	0.832	0.936
Pancreas	91.3	80.2	85.8	80.6	1.783	**0.027**	0.481	0.420
Spinal cord	25.4	25.0	26.4	24.9	0.336	0.144	0.513	0.393
Spleen	165.7	148.1	168.6	142.6	3.344	**0.002**	0.851	0.535
Testicles	132.46	123.82	136.06	128.38	2.832	0.157	0.475	0.933
Thymus	57.98	49.25	59.27	46.87	1.642	**0.002**	0.870	0.580
Thyroid glands	10.75	8.71	12.38	8.14	0.575	**0.009**	0.651	0.345
Tongue	210.37	197.64	210.92	197.27	3.456	0.063	0.990	0.947
Urinary bladder	15.41	13.63	16.46	19.19	0.959	0.805	0.092	0.246

Values are shown as LS means. The bold font indicates *p* < 0.05. ^#^ Data were recorded for 15 weeks ending with the slaughtering of the first bulls. ADG = average daily gain; AMPA = aminomethylphosphonic acid (degradation product of glyphosate); BW = body weight; CFP = concentrate feed proportion; CON = control; DMI = dry matter intake; GLY = glyphosate; HC = high number of concentrates (2.5–5 kg/animal/d); LC = low CFP (1 kg/animal/d); ME = metabolizable energy; PSEM = pooled standard error of means.

**Table 4:** Serum metabolites in German Holstein bulls fed with or without glyphosate-contaminated rations and varying concentrate feed proportions for 15 weeks.

		Experimental Group					*p*-Value						
	Week	CON_HC_ (*n* = 12)	CON_LC_ (*n* = 12)	GLY_HC_ (*n* = 11)	GLY_LC_ (*n* = 12)	PSEM	GLY	CFP	*t*	GLY×CFP	GLY×*t*	CFP×*t*	GLY× CFP×*t*
Albumin [g/L]	0	30.07	29.73	30.27	29.71	0.160	0.382	**<0.001**	**<0.001**	0.057	0.696	0.182	0.199
	7	29.62	27.28	28.44	27.43								
	15	33.32	30.33	31.62	31.19								
Total protein [g/L]	0	50.37	49.98	50.55	50.33	0.211	0.959	**<0.001**	**<0.001**	0.604	**0.018**	**0.003**	0.965
	7	54.14	52.23	52.15	50.95								
	15	53.42	49.32	54.50	50.83								
Total bilirubin [µmol/L]	0	7.91	7.63	7.87	7.71	0.097	0.369	**0.002**	0.945	0.927	0.773	**0.001**	0.364
	7	7.47	8.23	7.53	7.62								
	15	7.18	8.50	6.58	8.56								
Triglycerides [mmol/L]	0	0.19	0.21	0.18	0.19	0.004	0.085	0.467	**<0.001**	0.405	**0.039**	**0.014**	0.422
	7	0.13	0.08	0.15	0.12								
	15	0.17	0.15	0.19	0.21								
Alkaline phosphatase [µkat/L]	0	4.52	4.48	4.55	4.56	0.063	0.783	0.060	**<0.001**	0.131	0.948	0.352	0.622
	7	4.68	4.13	4.36	4.41								
	15	5.33	4.61	5.14	4.94								
Aspartate aminotransferase (AST) [µkat/L]	0	0.96	0.94	0.98	0.92	0.013	0.660	0.155	**0.030**	0.288	**0.046**	0.992	0.296
	7	0.93	0.87	0.96	0.96								
	15	1.11	1.00	0.95	0.98								
γ-glutamyltransferase (GGT) [µkat/L]	0	0.23	0.24	0.22	0.23	0.006	0.389	0.375	0.069	0.208	0.805	0.448	0.590
	7	0.26	0.22	0.23	0.22								
	15	0.28	0.24	0.26	0.26								
Glutamate dehydrogenase (GLDH) [µkat/L]	0	0.28	0.36	0.34	0.21	0.060	0.699	**0.025**	0.688	0.995	0.815	0.283	0.805
	7	0.66	0.15	0.69	0.21								
	15	0.20	0.23	0.39	0.18								
Urea [mmol/L]	0	1.01	1.15	0.89	0.99	0.069	0.312	0.477	**<0.001**	0.060	0.998	0.071	0.146
	7	3.59	3.09	2.82	3.58								
	15	3.43	2.69	3.09	2.72								
Uric acid [µmol/L]	0	65.23	92.32	86.59	77.23	4.416	0.417	0.335	**<0.001**	0.851	0.155	0.144	0.063
	7	21.40	12.06	17.62	16.77								
	15	87.81	52.37	54.91	53.20								

Values are presented as LS means. The bold font indicates *p* < 0.05. If week 0 had a significant influence (*p* < 0.05) in a parameter, the respective value was used as covariate. AST = aspartate aminotransferase; CFP = concentrate feed proportions; CON = control; GGT = γ-glutamyltransferase; GLDH = glutamate dehydrogenase; GLY = glyphosate; HC = high CFP; LC = low CFP; PSEM = pooled standard error of means; *t* = experimental time.

**Table 5:** Hematological parameters of the red blood profile in German Holstein bulls fed with or without glyphosate-contaminated rations and supplemented varying concentrate feed proportions for 15 weeks.

		Experimental Group					*p*-Value						
	Week	CON_HC_ (*n* = 12)	CON_LC_ (*n* = 12)	GLY_HC_ (*n* = 11)	GLY_LC_ (*n* = 12)	PSEM	GLY	CFP	*t*	GLY× CFP	GLY×*t*	CFP×*t*	GLY× CFP×*t*
Erythrocytes [10^6^/µL]	0	8.18	8.22	8.17	8.24	0.048	0.420	**0.021**	**<0.001**	0.204	0.845	0.125	0.558
	7	9.34	9.22	9.46	8.86								
	15	8.26	8.01	8.27	7.75								
Mean corpuscular volume [fL]	0	39.87	39.86	39.85	39.87	0.081	0.079	**<0.001**	**<0.001**	0.329	0.446	**<0.001**	0.639
	7	42.74	41.73	42.79	42.53								
	15	43.90	42.22	44.25	42.75								
Hemoglobin [g/dL]	0	9.69	9.68	9.65	9.74	0.039	**0.002**	**0.019**	**<0.001**	0.976	**0.006**	**<0.001**	0.678
	7	11.10	11.22	10.47	10.67								
	15	12.32	11.65	12.28	11.42								
Mean corpuscular hemoglobin [pg]	0	11.86	11.84	11.85	11.85	0.049	0.211	0.462	**<0.001**	0.142	0.072	**0.002**	0.502
	7	11.97	12.22	11.23	12.08								
	15	14.99	14.55	14.94	14.75								
Mean corpuscular hemoglobin concentration [g/dL]	0	29.83	29.47	30.06	29.65	0.161	0.093	0.072	**<0.001**	0.431	**0.007**	**0.001**	0.633
	7	28.12	29.09	26.55	28.32								
	15	34.16	34.26	34.07	34.32								
Hematocrit [%]	0	32.57	32.63	32.38	32.77	0.201	0.721	**<0.001**	**<0.001**	0.495	0.979	**0.007**	0.735
	7	39.84	38.36	40.18	37.64								
	15	36.15	33.78	36.40	33.09								
Red cell distribution width [%]	0	18.55	18.61	18.69	18.60	0.051	0.629	0.249	**<0.001**	0.196	0.814	0.401	0.350
	7	18.42	18.20	18.35	18.51								
	15	17.27	16.67	16.95	16.91								
Platelets [10³/µL]	0	193.86	193.43	201.83	180.27	6.052	0.596	0.902	**<0.001**	0.881	0.866	0.316	0.701
	7	291.61	281.26	311.28	285.94								
	15	192.86	204.68	190.28	227.10								
Mean platelet volume [fL]	0	3.65	3.62	3.62	3.67	0.021	0.568	**0.001**	0.090	0.406	0.663	**0.029**	0.463
	7	3.78	3.63	3.83	3.55								
	15	3.81	3.64	3.97	3.63								
Platelet distribution Width [%]	0	14.24	14.41	14.19	14.35	2.91	0.940	**0.004**	**<0.001**	0.581	0.651	**0.003**	0.951
	7	14.49	14.13	14.69	14.20								
	15	15.73	15.20	15.76	15.07								

Values are presented as LS means. The bold font indicates *p* < 0.05. If week 0 had a significant influence (*p* < 0.05) in a parameter, the respective value was used as covariate. CFP = concentrate feed proportions; CON = control; GLY = glyphosate; HC = high CFP; LC = low CFP; PSEM = pooled standard error of means; *t* = experimental time.

**Table 6:** DNA damage indicators of fattening German Holstein bulls fed rations with or without glyphosate residues and different concentrate feed proportions after 15 weeks.

	Experimental Group					*p*-Value		
	CON_HC_ (*n* = 12)	CON_LC_ (*n* = 12)	GLY_HC_ (*n* = 11)	GLY_LC_ (*n* = 12)	PSEM	GLY	CFP	GLY×CFP
Tail DNA [%]	22.10	10.56	7.36	13.23	1.38	**0.034**	0.309	**0.003**
Olive tail moment	29.81	8.57	5.36	11.05	2.15	**0.014**	0.077	**0.003**

Values are presented as LSmeans. The bold font indicates *p* < 0.05. CFP = concentrate feed proportion; CON = control; GLY = glyphosate; HC = high CFP; LC = low CFP; PSEM = pooled standard error of the means.

**Table 7:** Reactive oxygen species production and phagocytic activity in polymorphonuclear leukocytes of fattening German Holstein bulls fed rations with or without glyphosate residues and different concentrate feed proportions for 15 weeks. Presented are the proportions (%) and the mean fluorescence intensities (MFI) of TPA stimulated rhodamine positive cells (R123+) as well as the phagocytic polymorphonuclear leukocytes.

		Experimental Group					*p*-Value						
	Week	CON_HC_ (*n* = 12)	CON_LC_ (*n* = 12)	GLY_HC_ (*n* = 11)	GLY_LC_ (*n* = 12)	PSEM	GLY	CFP	*t*	GLY×CFP	GLY×*t*	CFP×*t*	GLY×CFP×*t*
Stimulated R123+ [%]	0	98.90	99.58	98.20	98.20	0.296	0.947	0.832	**<0.001**	0.186	0.346	0.869	0.293
	7	96.90	91.45	93.68	96.62								
	15	99.59	99.70	99.30	99.73								
Stimulated R123+ [MFI × 10^3^]	0	58.63	59.45	57.83	60.14	0.704	0.651	0.395	**<0.001**	0.511	0.935	0.853	0.324
	7	40.08	39.92	38.77	39.49								
	15	55.79	60.27	58.60	56.26								
Phagocytic PMN [%] ^#^	0	78.07	80.22	79.83	82.04	1.173	0.724	0.071	**0.005**	0.422	0.180	**0.004**	0.802
	7	86.66	86.00	83.70	80.74								
	15	82.13	74.93	84.99	73.42								
Phagocytic PMN [MFI × 10^3^] ^#^	0	81.19	82.38	81.11	82.00	1.133	0.590	0.142	**<0.001**	0.084	0.789	**0.040**	0.165
	7	101.52	75.40	75.15	83.24								
	15	109.57	83.76	100.67	95.01								

Values are presented as LS means. The bold font indicates *p* < 0.05. If week 0 had a significant influence (*p* < 0.05) in a parameter, the respective value was used as covariate. ^#^ Data of only 5–6 animals/group were collected at week 0 and 7; CFP = concentrate feed proportion; CON = control; GLY = glyphosate; HC = high CFP; LC = low CFP; PSEM = pooled standard error of the mean; *t* = experimental time; TPA = 12-O-tetradecanoylphorbol-13-acetate.

**Table 8:** Apoptotic polymorphonuclear leukocytes (PMN) and peripheral blood mononuclear blood cells (PBMC) of fattening German Holstein bulls fed rations with or without glyphosate residues and different concentrate feed proportions after 15 weeks.

	Experimental Group					*p*-Value		
	CON_HC_ (*n* = 12)	CON_LC_ (*n* = 12)	GLY_HC_ (*n* = 11)	GLY_LC_ (*n* = 12)	PSEM	GLY	CFP	GLY×CFP
Early apoptotic PMN [%]	3.72	4.32	3.2	6.81	0.62	0.452	0.114	0.254
Late apoptotic PMN [%]	0.65	0.63	0.88	0.90	0.08	0.126	0.990	0.921
Early apoptotic PBMC [%]	4.24	4.80	4.61	4.87	0.27	0.685	0.449	0.784
Late apoptotic PBMC [%]	4.83	4.84	5.12	5.40	0.30	0.476	0.812	0.816

Values are presented as LSmeans. CFP = concentrate feed proportion; CON = control; GLY = glyphosate; HC = high CFP; LC = low CFP; PBMC = peripheral blood mononuclear blood cells; PMN = polymorphonuclear leukocytes; PSEM = pooled standard error of the mean.

**Table 9:** Normalized mRNA levels of genes of interest in blood leukocytes of bulls treated with or without glyphosate-contaminated feed in the context of varying concentrate feed proportions after 15 weeks.

	Experimental Group					*p*-Value		
	CON_HC_ (*n* = 12)	CON_LC_ (*n* = 12)	GLY_HC_ (*n* = 11)	GLY_LC_ (*n* = 12)	PSEM	GLY	CFP	GLY×CFP
*BCL2*	0.89	0.82	0.98	0.94	0.012	**0.042**	0.249	0.669
*BRCA1*	0.88	0.91	0.98	0.99	0.012	0.083	0.712	0.852
*CASP3*	0.98	0.94	1.09	0.97	0.014	0.317	0.232	0.572
*CDKN1A*	0.81	0.61	0.76	0.66	0.016	0.965	**0.010**	0.340
*GADD45A*	0.71	0.71	0.80	0.73	0.016	0.275	0.568	0.527
*HSPA1A*	0.75	0.62	0.89	0.78	0.021	**0.043**	0.093	0.797
*NRF2*	0.86	0.74	0.90	0.76	0.010	0.477	**0.002**	0.915
*OGG1*	0.90	0.94	1.02	1.05	0.013	0.055	0.624	0.886
*PARP1*	0.86	0.79	1.02	0.90	0.012	**0.008**	0.051	0.734
*RAD51*	0.73	0.75	0.65	0.77	0.012	0.403	0.101	0.209
*SOD2*	0.88	0.67	0.91	0.69	0.013	0.670	**<0.001**	0.904
*TP53*	0.88	0.84	1.09	1.02	0.013	**0.002**	0.394	0.822
*XRCC5*	0.77	0.76	0.90	0.88	0.014	**0.024**	0.812	0.883

Values are presented as back-transformed LSmeans. The bold font indicates *p* < 0.05. *BCL2* = BCL2 apoptosis regulator; *BRCA1* = BRCA1 DNA repair associated; *CASP3* = Caspase 3; *CDKN1A* = cyclin dependent kinase inhibitor 1A; CFP = concentrate feed proportion; CON = control; *GADD45A* = growth arrest and DNA damage inducible alpha; GLY = glyphosate; HC = high CFP; *HSPA1A* = heat shock protein family A (Hsp70) member 1A; LC = low CFP; *NRF2* = nuclear factor erythroid 2–related factor 2; *OGG1* = 8-oxoguanine DNA glycosylase; *PARP1* = poly(ADP-ribose) polymerase 1; *RAD51* = RAD51 recombinase; *SOD2* = superoxide dismutase 2; *TP53* = tumor protein p53; *XRCC5* = X-ray repair cross complementing 5.

## Data Availability

Data in this manuscript were collected and managed in accordance with the data management policy of the FLI. Raw data for statistical analyses and supplemental data are available at Zenodo (DOI: 10.5281/zenodo.7861978).

## Appendix A

**Table A1:** Overview of numbers of animals used for statistical analyses of parameters, when differing from full data set (CON_HC_: *n* = 12; CON_LC_: *n* = 12; GLY_HC_: *n* = 11; GLY_LC_: *n* = 12).

Parameter	Experimental Week	Group	*n*
Albumin [g/L]	7	CON_LC_	11
Glucose [mmol/L]	7	CON_LC_	11
Cholesterol [mmol/L]	7	CON_LC_	11
AST [µkat/L]	7	CON_LC_	11
		GLY_LC_	11
GGT [µkat/L]	7	CON_LC_	11
GLDH [µkat/L]	0	GLY_LC_	11
	7	CON_LC_	10
	15	CON_HC_	11
		CON_LC_	10
Triglycerides [mmol/L]	7	CON_LC_	11
Urea [mmol/L]	7	CON_LC_	11
Uric acid [µmol/L]	7	CON_LC_	11
AP [µkat/L]	7	CON_LC_	11
Monocytes (%)	0	CON_HC_	11
Platelet distribution width	15	CON_HC_	11
		CON_LC_	11
Basal R123+ (%)	0	CON_LC_	11
	7	CON_LC_	11
		GLY_LC_	11
	15	CON_LC_	11
Stimulated R123+ (%)	0	CON_LC_	11
	7	CON_HC_	10
		CON_LC_	10
		GLY_HC_	10
		GLY_LC_	9
	15	CON_LC_	11
Basal R123+ (MFI)	0	CON_LC_	11
	7	CON_LC_	11
		GLY_LC_	11
	15	CON_LC_	11
Stimulated R123+ (MFI)	0	CON_LC_	11
	7	CON_HC_	10
		CON_LC_	10
		GLY_HC_	10
		GLY_LC_	9
	15	CON_LC_	11
CD4 (%)	15	CON_HC_	11
		CON_LC_	11
		GLY_LC_	10
CD4+ (MFI)	15	CON_HC_	11
		CON_LC_	11
		GLY_LC_	10
CD8+ (%)	15	CON_HC_	11
		CON_LC_	11
		GLY_LC_	10
CD8+ (MFI)	15	CON_HC_	11
		CON_LC_	11
		GLY_LC_	10
Phagocytotic PMN (%)	0	CON_HC_	6
		CON_LC_	6
		GLY_HC_	7
		GLY_LC_	5
	7	CON_HC_	7
		CON_LC_	7
		GLY_HC_	7
		GLY_LC_	7
	15	CON_HC_	11
		CON_LC_	11
		GLY_LC_	11
Phagocytotic PMN (MFI)	0	CON_HC_	6
		CON_LC_	6
		GLY_HC_	7
		GLY_LC_	5
	7	CON_HC_	7
		CON_LC_	7
		GLY_HC_	7
		GLY_LC_	7
	15	CON_HC_	11
		CON_LC_	11
		GLY_LC_	9
Early apoptotic PMN (%)	15	CON_LC_	11
Late apoptotic PMN (%)	15	CON_LC_	11
Early apoptotic PBMC (%)	15	CON_LC_	11
Late apoptotic PBMC (%)	15	CON_LC_	11
SOD (U/g HGB)	15	CON_LC_	11
Gpx (mU/g HGB)	15	CON_LC_	11

CFP = concentrate feed proportion; CON = control; GLY = glyphosate; Gpx = glutathione peroxidase; HC = high CFP; LC = low CFP; MFI = mean fluorescence intensity; PBMC = peripheral blood mononuclear cells; PMN = polymorphonuclear leukocytes; SOD = superoxide dismutase.

**Table A2:** Data statistics of serum metabolites in German Holstein bulls fed with or without glyphosate-contaminated rations and varying concentrate feed proportions for 15 weeks.

		*p*-Value						
	PSEM	GLY	CFP	*t*	GLY×CFP	GLY×*t*	CFP×*t*	GLY×CFP×*t*
Glucose [mmol/L]	0.024	0.296	0.228	**<0.001**	0.763	0.363	**<0.001**	0.828
NEFA [µmol/L]	0.007	0.802	**0.021**	**<0.001**	0.996	0.759	**0.016**	0.645
BHB [mmol/L]	0.01	**0.011**	0.053	0.848	0.236	**0.007**	**<0.001**	0.275
Cholesterol [mmol/L]	0.026	**0.032**	0.398	**<0.001**	0.677	**0.027**	0.405	**0.023**

The bold font indicates *p* < 0.05. BHB = β-hydroxybutyrate; CFP = concentrate feed proportion; GLY = glyphosate; NEFA = non-esterified fatty acids; PSEM = pooled standard error of means; *t* = experimental time.

**Table A3:** Data statistics of ferric reducing ability of plasma (FRAP) in German Holstein bulls fed with or without glyphosate-contaminated rations and varying concentrate feed proportions for 15 weeks.

			*p*-Value					
	PSEM	GLY	CFP	*t*	GLY×CFP	GLY×*t*	CFP×*t*	GLY×CFP×*t*
Fe^2+^ [µmol/L]	0.026	0.727	0.186	**<0.001**	0.379	0.414	**0.001**	**0.008**

The bold font indicates *p* < 0.05. CFP = concentrate feed proportion; GLY = glyphosate; PSEM = pooled standard error of means; *t* = experimental time.

**Table A4:** Data statistics of blood cell populations in German Holstein bulls fed with or without glyphosate-contaminated rations and varying concentrate feed proportions for 15 weeks.

		*p*-Value						
	PSEM	GLY	CFP	*t*	GLY×CFP	GLY×*t*	CFP×*t*	GLY×CFP×*t*
Leukocytes [10^3^/L]	0.089	0.477	**<0.001**	0.023	0.122	0.515	0.072	0.520
Lymphocytes [%]	0.349	0.817	0.073	**<0.001**	0.470	0.473	0.527	0.131
Monocytes [%]	0.075	0.679	0.471	0.864	0.296	0.582	0.483	0.449
Granulocytes [%]	0.577	0.910	**0.037**	**0.042**	0.198	0.828	0.928	0.815
Eosinophils [%]	0.488	0.472	0.631	0.069	0.397	0.733	0.953	0.657

The bold font indicates *p* < 0.05. CFP = concentrate feed proportion; GLY = glyphosate; PSEM = pooled standard error of means; *t* = experimental time.

**Table A5:** Data statistics of relative proportions and mean fluorescence intensity (MFI) of CD4+ and CD8+ cells of total peripheral blood mononuclear cells in German Holstein bulls fed with or without glyphosate-contaminated rations and varying concentrate feed proportions for 15 weeks.

		*p*-Value						
	PSEM	GLY	CFP	*t*	GLY× CFP	GLY×*t*	CFP×*t*	GLY×CFP×*t*
CD4+ [%]	0.168	0.127	0.360	**<0.001**	0.187	**0.043**	0.388	0.051
CD8+ [%]	0.161	0.070	0.479	**<0.001**	0.091	0.100	0.906	**0.003**
CD4+ [MFI]	0.025	0.289	0.520	**<0.001**	0.345	0.245	0.533	0.049
CD8+ [MFI]	0.530	**<0.001**	**0.005**	**<0.001**	0.058	**0.009**	**0.044**	**<0.001**

The bold font indicates *p* < 0.05. CFP = concentrate feed proportion; GLY = glyphosate; MFI = mean fluorescence intensity; PSEM = pooled standard error of means; *t* = experimental time.

**Table A6:** Data statistics of reactive oxygen species production in polymorphonuclear leukocytes of German Holstein bulls fed with or without glyphosate-contaminated rations and varying concentrate feed proportions for 15 weeks.

		*p*-Value						
	PSEM	GLY	CFP	*t*	GLY×CFP	GLY×*t*	CFP×*t*	GLY×CFP×*t*
Basal R123+ [%]	0.486	0.652	0.880	**<0.001**	0.254	0.118	0.087	0.231
Basal R123+ [MFI × 10^3^]	0.519	0.033	0.244	**<0.001**	0.955	0.083	**<0.001**	**0.002**

The bold font indicates *p* < 0.05. CFP = concentrate feed proportion; GLY = glyphosate; MFI = mean fluorescence intensity; PSEM = pooled standard error of means; *t* = experimental time
